# Phylogeny of the SNARE vesicle fusion machinery yields insights into the conservation of the secretory pathway in fungi

**DOI:** 10.1186/1471-2148-9-19

**Published:** 2009-01-23

**Authors:** Nickias Kienle, Tobias H Kloepper, Dirk Fasshauer

**Affiliations:** 1 Research Group Structural Biochemistry, Department of Neurobiology, Max-Planck-Institute for Biophysical Chemistry, Am Fassberg 11, 37077 Göttingen, Germany

## Abstract

**Background:**

In eukaryotic cells, directional transport between different compartments of the endomembrane system is mediated by vesicles that bud from a donor organelle and then fuse with an acceptor organelle. A family of integral membrane proteins, termed soluble N-ethylmaleimide-sensitive factor attachment receptor (SNARE) proteins, constitute the key machineries of these different membrane fusion events. Over the past 30 years, the yeast *Saccharomyces cerevisiae *has served as a powerful model organism for studying the organization of the secretory and endocytic pathways, and a few years ago, its entire set of SNAREs was compiled.

**Results:**

Here, we make use of the increasing amount of genomic data to investigate the history of the SNARE family during fungi evolution. Moreover, since different SNARE family members are thought to demarcate different organelles and vesicles, this approach allowed us to compare the organization of the endomembrane systems of yeast and animal cells. Our data corroborate the notion that fungi generally encompass a relatively simple set of SNARE proteins, mostly comprising the SNAREs of the proto-eukaryotic cell. However, all fungi contain a novel soluble SNARE protein, Vam7, which carries an N-terminal PX-domain that acts as a phosphoinositide binding module. In addition, the points in fungal evolution, at which lineage-specific duplications and diversifications occurred, could be determined. For instance, the endosomal syntaxins Pep12 and Vam3 arose from a gene duplication that occurred within the Saccharomycotina clade.

**Conclusion:**

Although the SNARE repertoire of baker's yeast is highly conserved, our analysis reveals that it is more deviated than the ones of basal fungi. This highlights that the trafficking pathways of baker's yeast are not only different to those in animal cells but also are somewhat different to those of many other fungi.

## Background

**Figure 1:**
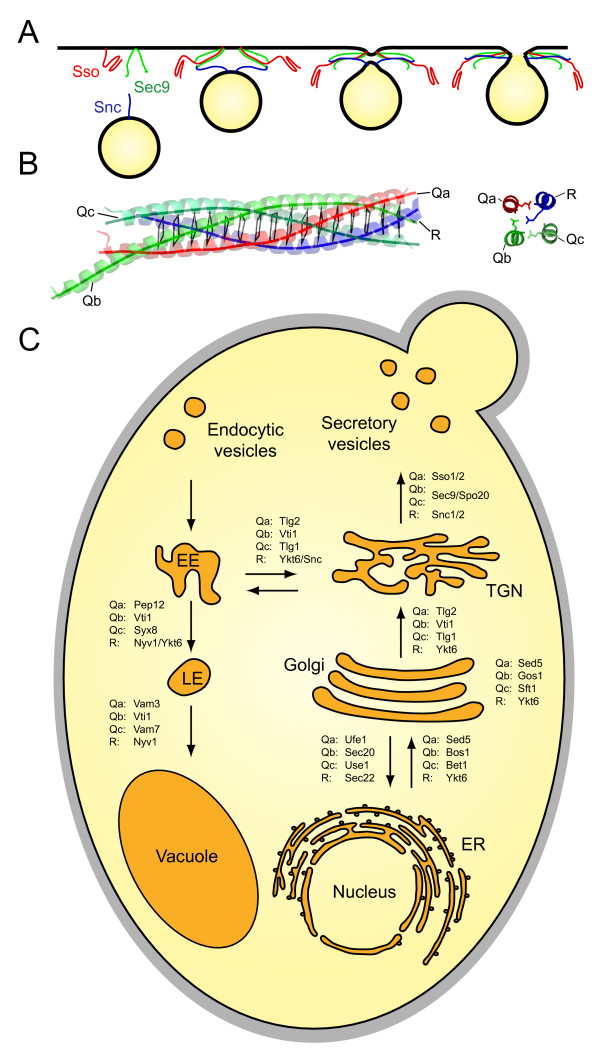
**SNARE proteins are key factors in vesicle trafficking in yeast**. A) Current model of the role of SNARE proteins in vesicle fusion. Heterologous sets of SNARE proteins, here exemplified by the secretory set of SNAREs, assemble in a zipper-like fashion into tight complexes between two membranes, in effect initiating fusion. B) Architecture of the archetypical four-helix bundle SNARE complex structure [91]. The structure of the neuronal SNARE complex is shown as ribbon diagram on the left (blue, red and green for synaptobrevin 2, syntaxin 1a and SNAP-25a, respectively). The layers in the core are indicated by virtual bonds between the corresponding C⟨ positions. The structure of the central 0-layer is shown in detail on the right [91]. According to this unusual hydrophilic layer in the center of the complex, SNAREs have been categorized as either Q- or R-SNAREs depending on whether they contribute a conserved glutamine or arginine, respectively, to this layer. The three Q-SNAREs are further subdivided into Qa-, Qb- and Qc-SNAREs based on the other sequences of their SNARE domains. These four basic types correspond to the four different helices of the canonical SNARE complex architecture [91-94]. Hence, functional complexes are composed of four different helices, each belonging to one of the four main groups ("QabcR" composition) [21,40,74,95,96]. C) Schematic outline of the vesicle trafficking pathways and tentative assignement of the involved sets of SNARE proteins of baker's yeast. It should be kept in mind, however, that the assignment of some SNAREs to certain trafficking steps, in particular of the R-SNAREs, is still debated. Note that baker's yeast has two endosomal syntaxins, Pep12 and Vam3, that are thought to be involved in consecutive trafficking steps towards the vacuole, whereas other fungi only have one.

Over the last decades, the organization of the secretory and endocytic pathways has been studied in a variety of different eukaryotic organisms. These studies have revealed that the principal organization of the endomembrane system and the molecular machineries involved in vesicular trafficking are conserved among all eukaryotes. In general, the transport between different intracellular organelles is mediated by cargo-laden vesicles that bud from a donor and then specifically fuse with an acceptor compartment. Key players during the final fusion step are the so-called SNARE proteins. These proteins are a large family of small cytoplasmically orientated membrane proteins that are typically tail-anchored. Their key characteristic is the so-called SNARE domain, an extended stretch of heptad repeats that is usually connected to a single transmembrane domain by a short linker. SNARE domains of heterologous sets of SNARE proteins have the ability to assemble into tight, parallel four-helix bundle complexes. It is thought that complex formation between SNARE proteins from opposing membranes provide the energy that drives membrane fusion (reviewed in [1-4], Fig. 1A &1B).

Although the concept of SNARE-mediated membrane fusion emerged in the 1990s [5-7], several SNARE proteins had been discovered earlier. The field of membrane trafficking was greatly inspired by ingenious genetic screens in the single-cell fungi *Saccharomyces cerevisiae *for mutants with defects in the secretory pathway in the late 1970s [8,9]. In these mutants, secretion was blocked at higher temperatures. These so-called *SEC *mutants accumulate secretory proteins at the point in the secretory pathway that is blocked and often, they could be distinguished by their phenotype. In the following years, it became clear that *SEC *genes generally encode for key components of the machineries that mediate trafficking of a transport vesicle, including a few SNARE proteins. All at once, straightforward genetic tools had catapulted baker's yeast to the front row for studying vesicle trafficking pathways. In fact, other genetic screens for mutants showing defects in other transport processes (reviewed in [10-13]) have turned up novel yeast SNAREs. The last yeast SNARE was tracked down in 2003, yielding an overall repertoire of 24 different SNARE genes in baker's yeast [14] [see Additional file 1]. For most trafficking steps in baker's yeast, distinct units of four interacting SNARE domains have now been assigned ([1,2,14,15], Fig. 1C). However, not all details of the trafficking routes in yeast are resolved. Generally, different SNARE family members are localized on distinct organelles and vesicles that demarcate particular membranes. However, it is becoming clear that some trafficking steps cannot be identified by a unique set of SNAREs, since some SNAREs are involved in more than one trafficking step. In addition, they may participate in different SNARE complexes (for a discussion, see [16,17]). To appraise *S. cerevisiae *as model organism for studying vesicle trafficking pathways better, it is also necessary to understand the limits of the organism, since baker's yeast appears to be a secondarily reduced organism. For example, *S. cerevisiae *has only a limited ability to produce hyphae (long branching multicellular filaments). The capability to produce polarized hyphae, however, is a hallmark of the fungal kingdom, allowing them to colonize and exploit new substrates efficiently. In addition, whereas most fungi are multicellular organisms, *S. cerevisiae *most of the times grows as single cells that reproduce asexually. It thus seems possible that adaptation to a relatively simple lifestyle was accompanied by a degeneration of the intracellular trafficking itinerary together with the involved machineries.

A comparison of the SNARE gene repertoires of *S. cerevisiae *with that of five other fungal species, including three species of filamentous fungi, revealed that, in general, the members of the SNARE family are largely conserved in fungi [18]. This notion has been corroborated by subsequent inspections of the SNARE repertoire of few other fungal species [19,20]. Together, these studies put forward that the yeast lifestyle did not entail a radical change in the intracellular trafficking pathways. The bioinformatic strategies used to identify the homologs of the yeast SNAREs, however, did not provide a universal classification scheme. Consequently, it was impossible to compare the SNARE sets of different fungi with each other in an unambiguous manner, let alone comparing them with SNARE sets from other eukaryotes like animals. Hence it remains unclear whether SNARE types, which are possibly linked to a novel transport step, evolved or degenerated in particular fungal lineages.

Fungi are thought to be more closely related to animals than to plants and, currently, are placed with animals and several protistan taxa into the monophyletic group of Opisthokonta. Notably, morphological observations show that the appearance of analogous intracellular organelles such as the vacuoles/lysosomes and the Golgi apparatus are markedly different between yeast and animal cells. It is unclear, however, whether these differences are reflected in their respective SNARE inventory. Recently, using Hidden Markov Models (HMM) we have established a precise classification for all eukaryotic SNARE proteins [21]. Based on this classification, we were able to deduce that the emergence of multicellularity in animals went along with an expansion of the set of SNARE proteins, in particular the expansion of the SNAREs involved in endosomal trafficking [22].

In order to analyze the differences between the SNARE repertoires of fungi and animals and the evolutionary changes in the SNARE inventory of different fungal lineages, we have collected and classified the SNAREs from a broad range of different fungi according to our HMMs, making use of the substantial number of available genomic sequences. To identify specification and duplication events, and to pinpoint the differences between the set of SNARE proteins in fungi and animals, we used phylogenetic information to reveal the history of SNARE genes. Afterwards, we mapped features of the fungal SNARE evolution onto the species tree.

## Methods

### Sequences

Recently, we published a classification for SNARE sequences, based on more than 2000 protein sequences from 145 eukaryotic species, including 718 sequences from 51 species of fungi. We were able to identify twenty basic subtypes within the SNARE protein family and developed specific as well as sensitive HMMs for each subtype [21,23]. We used these models to identify SNARE proteins in fungal genomes that became available recently. The models yielded 1221 additional sequences from the nr-database at NCBI and various genome projects (DOE Joint Genome Institute), Baylor College of Medicine, J. Craig Venter Institute and Broad Institute) and several EST databases (NCBI Expressed Sequence Tags database, Fungal Genomics Project, and Taxonomical Broad EST Database) [24]. The newly found protein sequences underwent a visual verification process to remove redundancy and misassembled sequences. Overall, our data contained 1536 unique SNARE sequences from 123 fungal species. The dataset was incorporated into the SNARE database [21] and can be accessed on the project homepage . The SNARE sets of all fungal species with complete genomes sequenced can be found in [Additional file 2].

For a more detailed inspection of the evolutionary changes within fungi, we made use of the observation that within a subtype, sequence regions besides the SNARE motif were also well conserved. Consequently, we constructed alignments of the entire sequences for each subgroup using muscle [25]. Sites with more than 25 percent gaps were removed from the alignments. In addition, we excluded all sequences which contained more than 50 percent gaps. These alignments are also available via the supplementary section of our projects homepage. Based on these alignments, we constructed phylogenetic trees for each of the fungal SNAREs [see Additional file 3]. We also generated sequence alignments in which we graphically incorporated information about the domain structure based on the available high resolution structure information [see Additional file 4].

### Phylogeny

The phylogenetic reconstruction was composed of two different analytical approaches and was basically done as described in [21]. The first approach used IQPNNI (Important Quartet Puzzling and Nearest Neighbor Interchange) [26] to construct phylogenetic trees from the curated alignments. We used a gamma distribution as a model for rate heterogeneity with four rate categories for the estimation of the gamma distribution parameter. The proportion of invariable sites was estimated from the data and the Jones, Taylor and Thornton (JTT-) distance matrix [27] served as a substitution matrix. We used the stopping rule of IQPNNI, but the calculation had to run for at least the suggested number of iterations. The default values were used for the remaining parameter. In addition, Likelihood-Mapping was applied to determine the confidence of the edges in the calculated trees. The second approach used the phylip package [28] to apply a distance-based bootstrap analysis with 1000 replicates to each of the curated alignments. Standard settings were used for seqboot, the JTT distance matrix and also a gamma distribution (with parameter approximation from tree-puzzle) for protdist, as were standard options for neighbour. If required, the random seed was set to nine. We used the almost unbiased (AU) test [29] to address the systematically biased bootstrap values. We obtained the sitewise log-likelihoods needed for the AU test using a modified version of phyml [30] and the test was performed using consel [31]. The reconstructed IQPNNI trees served as starting points to join the results of both calculations. The inner edges of the trees were labeled with their Likelihood-Mapping and corrected bootstrap support values. We provide Nexus files for each reconstructed tree on our project homepage [see Additional file 3]. The trees can be interactively explored using, for example, SplitsTree 4 [32].

To gain initial insights on the placement of fungi within the Opisthokonta, especially the basal fungal species *Batrachochytrium dendrobatidis *and *Blastocladiella emersonii*, we first performed an extensive phylogenetic analysis based only on the sequence of the SNARE domain. This analysis was carried out to ensure that the evolutionary history of the individual SNARE groups indeed reflects the monophyletic origin of fungi. For this analysis, we used all fungal species together with the choanoflagellate *Monosiga brevicollis*, the slime mold *Dictyostelium discoideum*, and ten selected animal species [see Additional file 5]. We also included the SNAREs found in the animal pathogen *Encephalitozoon cuniculi*, which belongs to the Microsporidia. In earlier times, this clade was considered to be of protist origin, but it is now believed to have diverged from early fungi. However, we purposefully did not include their sequences into the general phylogenetic analysis, as *E. cuniculi *appears to have a markedly reduced set of SNARE proteins [21] with clearly deviating sequences, making them sensitive to long branching artefacts. The reconstructed phylogenetic trees demonstrate that fungi (including the basal species) are well separated from other opisthokonts (the phylogenetic trees can be downloaded from our projects homepage). With this corroborated, we then performed a more detailed phylogenetic analysis of the SNAREs in fungi using the entire sequence.

## Results and discussion

In order to catalog the SNARE inventory of fungi, we increased our previous collection [21] with newly published genomes, and complemented this set with ESTs from different fungal species, obtaining an overall set of more than 1500 SNARE sequences from 123 fungal species. The dataset comprises about 70 species with nearly complete sets of SNAREs [see Additional file 2]. All sequences and species used in this study can also be found in our SNARE database on our projects homepage . The collected sequences were then classified according to our previously established HMM models [21] that are based only on the highly conserved SNARE motif. The four main classes were then further subdivided into twenty distinct groups according to our HMM models. Each of these subgroups represents a distinct conserved trafficking step. For phylogenetic analysis of the fungal lineage, we aligned entire sequences for each subgroup, as evolutionary changes have usually occurred by paralogous expansion of particular SNARE types. Based on these alignments, we constructed phylogenetic trees for each of the SNARE types in fungi [see Additional file 3].

### Fungi contain a compact set of SNARE proteins

Overall, the fungal genomes examined in this work comprise a remarkably consistent and homologous set of SNARE proteins. In each species with a complete genome, we only found a little more than twenty different SNAREs, whereas in metazoans, for example species with more than 30 SNAREs are common. A marked expansion of the metazoan set of SNAREs occurred during the rise of multicellularity [22]. In earlier studies it was put forward that the development of multicellularity is generally linked to an expansion of the SNAREs [33-35]. In contrast to this idea, we usually only found members of the basic SNARE proteins to be present in each fungi species. This implies that fungi, although they also have evolved multicellularity, have a relatively unchanged set of SNAREs and have not significantly expanded the repertoire of the proto-eukaryotic cell.

It was not the objective of the present study to discuss the function of each SNARE protein coded for in fungal genomes in detail, as the sequence information alone is insufficient for recognizing the exact trafficking step the protein is involved in [see Additional file 6]. Instead, we will mainly focus on highlighting the idiosyncrasies that we came across. Notably, the SNARE proteins involved in secretory trafficking routes between the endoplasmatic reticulum (ER), the Golgi apparatus (GA) and the trans-Golgi network (TGN) were generally present as singletons and were highly conserved in all fungi. Noticeable changes affected the set of SNARE proteins involved in secretion and in the endosomal/vacuolar trafficking pathway.

An interesting observation is that the set of SNARE proteins in fungi is very similar to the set of the single-cell choanoflagellate *Monosiga brevicollis*, which is thought to be closely related to animals. In contrast to *M. brevicollis*, lower animals possess an enlarged set of SNARE proteins [22]. A surprising aspect of the chytrids *Batrachochytrium dendrobatidis *and *Blastocladiella emersonii *is that they possess a SNARE that was classified as Qb.III.d-type [21]. SNAREs belonging to this subclass were first found in green plants and termed novel plant SNAREs (Npsn) [36]. Our previous analysis had shown that Npsn can be found in plants and several protist lineages, but not in choanoflagellates and animals [21]. The fact that basal fungi retained a *bona fide *Npsn suggests that late-diverging fungi have lost Npsn independently. Interestingly, the chytrids are the most primitive of the fungi; many are aquatic and possess a flagellum. Consistent with their more basal position, sequences from the chytrids *Batrachochytrium dendrobatidis *and *Blastocladiella emersonii *are usually placed close to the root of the fungal lineage in our analysis. These results suggest that Npsn was a member of the original SNARE repertoire of the eukaryotic cenancestor but was then lost in the choanoflagellate/animal and the fungal lineage.

Another protein family has been widely used as a marker for the different trafficking steps of the endomembrane system. These are Rab proteins, a family of small GTP-binding proteins that regulate different vesicular trafficking steps by cycling between active GTP and inactive GDP forms [37,38]. It has been observed that fungi contain a relatively small number of Rab proteins [39], whereas their number has expanded in several other lineages such as animals [37,40], land plants[41], and some protists [42-45]. It would therefore be interesting to investigate whether the pattern of duplications and diversifications found for the Rab family in fungi [39] correlates with that of the SNARE family [22].

### The SNARE protein Vam7 is a molecular "invention" of fungi

Our analysis shows that fungi contain three different SNAREs of the Qc.III-type: Tlg1, syntaxin 8 (Syx8) and Vam7 [46-49]. The choanoflagellate *M. brevicollis *and basal animals contain only two Qc.III-SNAREs, Syx6 and Syx8, which are homologous to Tlg1 and Syx8, respectively. Remarkably, we found Vam7 homologs to be present in all complete fungal genomes, even in the basal fungus *B. dendrobatidis*. However, no Vam7 homolog was discovered in other eukaryotes, suggesting that the Qc.III-SNARE Vam7 is a new innovation in fungi. Of note, this status of Vam7 had been recognized earlier by Yoshizawa and colleagues [19]. However, one should keep in mind that their analysis was based on an inferior cluster approach that led, from a biological point of view, to a number of wrongly assigned clusters. In addition, they have missed several well-known SNARE proteins. Although the SNARE motif of Vam7 is not greatly diverged, it remains unclear whether an ancestral Tlg1 or Syx8 gave rise to Vam7. Nonetheless, considering its unique domain structure, Vam7 is the most divergent SNARE protein in the repertoire of fungi. In contrast to most other SNARE proteins, Vam7 does not carry a transmembrane region at the C-terminal end of its SNARE motif. Instead, the protein seems to be anchored to a membrane by means of an N-terminal extension that harbors a Phox homology (PX) domain, which acts as phosphoinositide binding module. In fact, Vam7 has been shown to interact specifically with phosphatidylinositol 3-phosphate (PI(3)P) [50], a lipid that is specific for endosomal and vacuolar membranes. A large body of work carried out in yeast implies that Vam7 catalyzes homotypic vacuole fusion with its partner SNAREs, Vam3 (Qa.III.b), Vti1 (Qb.III.b) and Nyv1 (R.III) [11,13]. Interestingly, however, the homologous factor in the basidiomycete *Ustilago maydis*, termed yup1, was found to be present on vacuoles and rapidly moving endosomal organelles. Moreover, a temperature sensitive mutation in yup1 blocks sorting to vacuoles and a defect in endosomal recycling [51-53] suggests that the function of Vam7 is not restricted to homotypic vacuole fusion, a process that, as we will discuss below, may be restricted to Saccharomycotina.

### Changes in the endosomal and vacuolar set of SNARE proteins in Saccharomycotina

**Figure 2:**
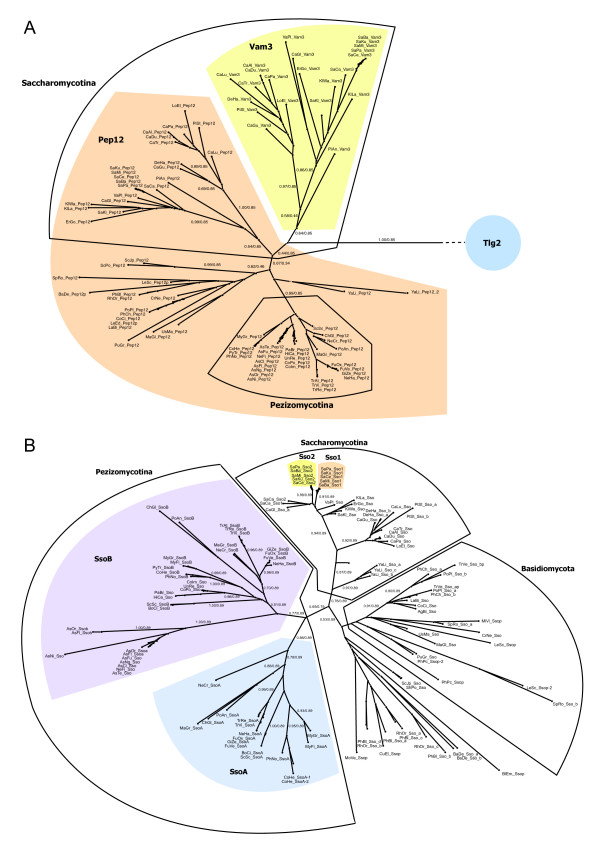
**Unrooted phylogenetic trees highlighting events of gene duplication and diversification in fungi**. A) The endosomal Qa-SNAREs (Qa.III.b-type) of fungi split into two major branches, Pep12 and Vam3, within the Saccharomycotina. The syntaxin involved in trafficking towards the TGN, Tlg2 (Qa.III.a-type) is shown as outgroup. B) The phylogenetic tree of secretory syntaxins (Qa.IV-type) reveals independent expansions in Pezizomycotina and in the *Saccharomyces senso-stricto *clade. The latter expansion probably occurred during a whole-genome duplication, during which the other secretory SNAREs were duplicated as well. Note that independent duplications of Sso genes occurred in other lineages as well. In each tree, the diverged SNARE types are shown by different colors. In addition, the major phylogenetic groups of fungi are indicated. The labels on the major branches represent the Likelihood Mapping (left) and AU support values (right).

The yeast *S. cerevisiae *belongs to the Saccharomycotina clade, the members of which are sometimes referred to as hemiascomycetes. *S. cerevisiae *possesses two endosomal syntaxins (Qa.III.b-type), Pep12 and Vam3, which are believed to be involved in trafficking to late endosomes and to vacuoles, respectively. In agreement with the earlier assessment by Gupta and Heath [18], our phylogenetic tree shows that these two SNAREs have been duplicated in the lineage of Saccharomycotina only (Fig. 2A). In addition, our analysis also demonstrates that the duplication of ancestral Qa.III.b-SNARE into Pep12 and Vam3 is independent of the duplications of the endosomal syntaxins in animals, i.e. syntaxin 7, syntaxin 13, syntaxin 17 and syntaxin 20.

The bifurcation of an ancestral endosomal syntaxin within the Saccharomycotina clade into proteins with distinct roles, namely Pep12 and Vam3, may suggest that the specialized machinery involved in homotypic vacuole fusion arose only within the hemiascomycetes. Homotypic vacuole fusion is a process that occurs during cell division in baker's yeast. During budding, the few large vacuoles maintained in *S. cerevisiae *are fragmented. Multiple small vacuoles then segregate into the budding daughter cell and subsequently fuse again into larger vacuoles [54-56]. This phenomenon has been exploited by William Wickner and colleagues [11,13] to study the role of SNAREs and other factors in the fusion process. This process, however, does not take place in, for example, *S. pombe*, a yeast that recently was proposed to belong to a third ascomycetes class, the Archiascomycotina [57]. This suggests that homotypic vacuole fusion, described first in baker's yeast, is a process specific to hemiascomycetes. Notably, the two Qa.III.b-SNAREs of *Yarrowia lipolytica *are much less diverged. Consequently, it seems interesting to find out at which point in the evolution of the Saccharomycotina lineage the process of homotypic vacuole fusion evolved. In addition, the sets of proteins catalyzing trafficking to the vacuole (*VPS *genes) and maintaining a low vacuole copy number (*VAM*) genes have only partial functional overlap [11,12]. It would thus be interesting to investigate whether the factors specifically involved in the process of homotypic vacuole fusion are restricted to the Saccharomycotina.

Previously, it has been debated whether the R-SNARE involved in trafficking towards vacuoles in yeast, Nyv1, is indeed homologous to the animal R-SNARE Vamp7 (also referred to as TI-Vamp). In fact, the SNARE motif of Nyv1 from baker's yeast is only distantly related to Vamp7 in animals [58,59]; however, in our analysis, both clearly fall into the same group (R.III). It has even been thought that Nyv1 does not contain a canonical profilin-like N-terminal extension, often also referred to as longin domain [58,59]. A recent structural analysis has demonstrated that the N-terminal extension of Nyv1 indeed contains a profilin-like fold of this type [60], similar to the ones found in Sec22 (R.I) [61] and Ykt6 (R.II) [62]. In general, all fungi possess a Nyv1 homolog. The only fungal lineages that do not appear to contain a Nyv1 homolog are members of Schizosaccharomyces (*S. pombe *and *S. japonicus*). Our phylogenetic analysis shows that Nyv1 sequences are remarkably derived in Saccharomycetales, bringing to light why it has been difficult before to acknowledge Nyv1 as a *bona fide *endosomal R-SNARE. In *S. cerevisiae*, Nyv1 seems to interact only with the Q.III.b-type syntaxin Vam3 but not with Pep12, which is thought to interact preferentially with the R-SNARE Ykt6. Hence, it seems possible that Nyv1 and Vam3 coevolved in Saccharomycotina.

### The larger set of secretory SNAREs in *S. cerevisiae *can be attributed to a whole genome duplication

In contrast to the notion that baker's yeast is a reduced organism, no fungal genome inspected contained more SNARE genes than *S. cerevisiae *with its 26 different SNARE factors (this count includes the regulatory SNARE proteins Sro7 and Sro77). These 26 genes are clearly more than the 20/21 genuine SNAREs of the proto-eukaryotic cell. Two of the added SNAREs Vam7, a novel, fungi-specific SNARE, and Vam3, a Saccharomycotina-specific SNARE were discussed above. In addition, baker's yeast contains four closely related pairs of secretory SNARE proteins, Sso1 and Sso2 (Qa.IV), Snc1 and Snc2 (R.IV), Sec9 and Spo20 (Qbc.IV), and Sro7 and Sro77 (R.Reg). Our analysis demonstrates that these doubled set of secretory factors very probably arose from a well established whole genome duplication (WGD) in *S. cerevisiae *[57,63]. Indeed, we found the same duplicated set of SNARE proteins in the closely related species *S. paradoxus*, *S. mikatae*, *S. bayanus *and *S. kudriavzevii*, all of which are collectively referred to as *Saccharomyces senso-stricto *species (Fig. 2B).

Remarkably, some but not all of the same secretory SNARE genes were also found in duplicates in *S. castellii*, *Candida glabrata*, and *Vanderwaltozyma polyspora *(synonym *Kluyveromyces polysporus*). The ancestor of this group of species (referred to as *Saccharomyces senso-lato*) and of the *Saccharomyces sensu stricto *species are believed to have undergone the same WGD followed by major secondary reductions, while several closely related species like *Eremothecium gossypii *(synonym *Ashbya gossypii*), *Kluyveromyces waltii*, *Kluyveromyces lactis*, and *Saccharomyces kluyveri *have kept a single copy of the genome. These species are grouped with the ones that underwent a WGD as, "*Saccharomyces *complex"[64]. Indeed, we found only one copy of each of the secretory SNAREs in the latter species. Interestingly, the duplicates found in *S. castellii*, *C. glabrata *and *V. polyspora *usually split at clearly different positions in our phylogenetic trees as compared to the duplicates of the *Saccharomyces sensu stricto *species. This is particularly noticeable for the two Sec9 proteins from *S. castellii *and also from *V. polyspora *that are highly similar, whereas the two Qbc-SNAREs, Sec9 and Spo20, exhibit marked asymmetrical rates of evolution in the *Saccharomyces sensu stricto *species, pointing to a different fate of the duplicated secretory SNAREs in the two lineages. Similarly, the two Sso proteins of *C. glabrata *are clearly more diverged than the duplicated set of the *Saccharomyces sensu stricto *species. In fact, it has been proposed that these lineages must have diverged and adapted to different living conditions very soon after the WGD, so that the genes retained in duplicates diverged largely independently [65].

Although only about 14% of all duplicated genes were retained after the WGD, all duplicated SNARE genes are involved in the process of secretion in baker's yeast. This suggests that these factors presented a selective advantage for yeast. The selective advantage of the duplicated secretory set is not entirely clear, since so far, only subtle functional differences of the duplicated SNAREs have been found. As mentioned above, only one homologous pair, the two Qbc-SNAREs (i.e. SNAP-25 related factors, Sec9 and Spo20) is highly diverged in the *Saccharomyces sensu-stricto *species. Sec9 and Spo20 possess only approximately 37% identity, whereas the homologs of the secretory syntaxins (Sso1 and Sso2) and R-SNAREs (Snc1 and Snc2) are 79% and 74% identical, respectively. Whereas Sec9, particularly in Saccharomycotina, possesses a rather long N-terminal extension, this region was reduced in Spo20 [66]. Interestingly, Sec9 and Spo20 are specialized to different developmental stages in baker's yeast. Whereas Sec9 interacts with both secretory syntaxins and synaptobrevins in secretion during vegetative growth, the more deviated Spo20 is required only for sporulation. During sporulation in *S. cerevisiae*, the prospore membrane, which envelops each daughter nucleus during meiosis, is generated *de novo *adjacent to the spindle pole bodies by the secretory SNARE machinery [67]. Nevertheless, Sec9 and Spo20 show some partial functional redundancy during sporulation, because only the *SPO20/SEC9 *double mutants exhibit a complete loss of prospore membranes, while *SPO20 *mutants show a milder sporulation phenotype and *SEC9 *mutants have no sporulation phenotype [66-68]. Furthermore, the two secretory syntaxins Sso1 and Sso2 seem to have some functional differences, because only Sso1 seems to be necessary during sporulation [69,70].

### Degeneration of the tomosyn SNARE motif in Saccharomycotina

In our previous study, we established that tomosyn (R.Reg), a regulatory R-SNARE without a membrane anchor, is a member of the basic SNARE repertoire of the proto-eukaryotic cell. Structural studies on the yeast tomosyn homolog, Sro7, have demonstrated that this factor carries a large N-terminal domain composed of two consecutive seven-bladed β-propeller domains [71]. However, tomosyn cannot serve as a fusogenic R-SNARE because it does not have a transmembrane anchor at its C-terminal end [72-75]. In a molecular process that is not completely understood to date, tomosyn is believed to function in establishing polarized secretion by controlling the accessibility of the t-SNAREs acceptor complexes [71]. As mentioned above, in baker's yeast, two close homologs of tomosyn exist, Sro7 and Sro77 (55% identity) [76]. They are often confused with the factor Lethal giant larvae (Lgl), which has a function in establishing epithelial cell polarity in animals [77,78]. The main basis for this confusion is that Sro7 and Sro77, like Lgl, do not possess the canonical R-SNARE motif found in tomosyns. In fact, Lgl derived independently from tomosyn in animals [75,79], where it lost its SNARE domain [22]. Interestingly, the canonical R-SNARE motif of tomosyn is present in other fungal tomosyns [75,79]. Our current analysis demonstrates that the R-SNARE motif degenerated in the Saccharomycetales clade.

### Two markedly diverged secretory syntaxins within Pezizomycotina

Our analysis also demonstrates that secretory SNAREs, in particular the secretory syntaxin (Sso, Qa.IV), have been duplicated recurrently in several fungal lineages, but no clear pattern between duplications and lifestyle or taxonomical grouping has been detected. Usually, duplicated SNAREs are not very greatly diverged in fungi. However, as Gupta and Heath have already had pointed out, the sodariomycete *Neurospora crassa *contains two clearly diverged secretory syntaxins, nsyn1 and nsyn2 [18,80]. A similar set of diverged secretory syntaxins has been described recently in *Trichoderma reesei *[81], which also belongs to the Pezizomycotina. The Pezizomycotina are filamentous fungi, which are sometimes also called euascomycetes. Besides the Saccharomycotina, they are the second major subphylum of the Ascomycotina, which are defined by the production of a specialized structure, the ascus, that surrounds the meiotic spores. Most Pezizomycotina species are hyphal fungi. Hyphae grow at their tips, a process that involves highly polarized secretion of cell wall material. The two syntaxins of *T. reesei*, Sso1 and Sso2, were found to be involved in different secretory processes. Whereas Sso2 was found in the apical compartments of actively growing hyphae, Sso1 was found in older, non-growing hyphae. *In vivo *FRET studies also suggest that the two proteins exert their secretory function at spatially segregated areas of the plasma membrane [81]. Our analysis now shows that two diverged secretory syntaxins in *N. crassa *and *T. reesei *represent a split that arose within the lineage of Pezizomycotina (Fig. 2B).

Notably, some species of the Eurotiomycetes, a lineage of the Pezizomycotina including the genus *Aspergillus*, only possess one Sso, suggesting that their lineage split before the duplication of secretory syntaxins [82]. Some *Aspergillus *species, *A. oryzae *and *A. flavus*, do possess two different secretory syntaxins. However, this duplication appears to have occurred independently within the Eurotiomycetes. In order to avoid confusion between the well-known duplicated set of secretory syntaxins in *S. cerevisiae *(Sso1 and Sso2) we suggest to name these two clearly diverged secretory syntaxins in Pezizomycotina SsoA and SsoB.

We noted that the duplication of the secretory syntaxin was not accompanied by a duplication of the interacting SNARE partners of Sso, Snc and Sec9. These usually exist only as one copy in the respective species of Pezizomycotina, suggesting that, as shown for *T. reesei *[81], Snc and Sec9 can interact with two different syntaxins. Interestingly, Sec9 in a few Sodariomycetes, namely *Magnaporthe grisea*, *Neurospora crassa*, *Podospora anserina *and *Chaetomium globosum*, is clearly diverged.

### SNAREs recapitulate the phylogeny of fungi

**Figure 3:**
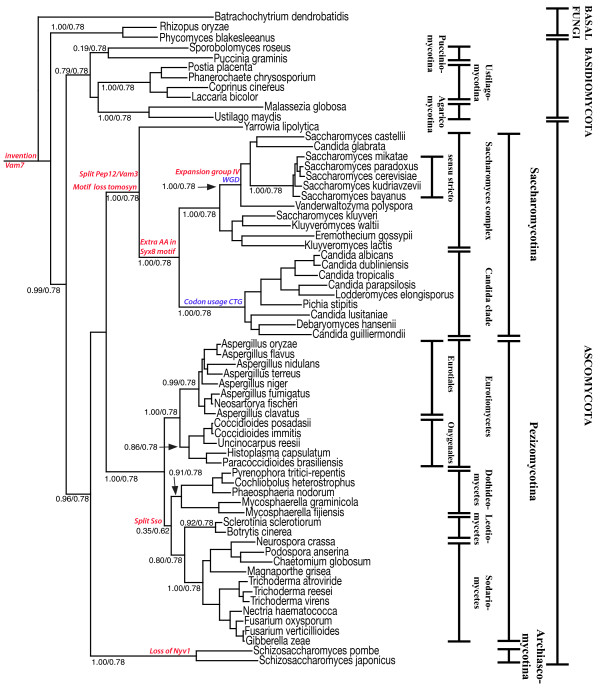
**Phylogenetic relationships based on concatenated SNARE sequences from 66 sequenced fungal genomes**. The major clades are named. Species that share the whole-genome duplication (WGD) [57,63] and those with the different code usage (CTG) [87,88] are indicated in blue, SNARE protein-derived synapomorphies are indicated in red. Support of the clades are represented by likelihood mapping values (left) and AU-support values (right). The best AU-support value returned by the bootstrap analysis was 0.78.

Multi-gene phylogenetic studies have started to change our view of the phylogenetic relationships within the fungi kingdom. In particular, the clades generated by those studies [57,63,64,82-86] differ markedly from previous classifications of yeasts that were usually defined mostly by morphological analysis and growth responses. We have already noted that the individual gene trees built from orthologous SNAREs generally recapitulate the newer classification. In fact, the sequences of species that belong to the same clade usually congregate in the individual trees. This indicates that SNARE proteins have diversified only slowly during evolution, but fast enough to reflect species evolution within the fungi kingdom. For a better resolution of species evolution, we concatenated the SNARE sequences that are present as singletons. The resulting phylogenetic tree agrees very well with the recently refined classification of the major fungal lineages (Fig. 3). The tree clearly resolves the major fungi phyla Basidiomycotina and Ascomycotina. The latter clearly split into two major subphyla, the Pezizomycotina and the Saccharomycotina, whereas the two schizosaccharomyces species sit outside the subphyla clades. Our data corroborate the notion that within the Pezizomycotina, the Eurotiomycetidae split first. As discussed above, the Eurotiomycetidae possess only one secretory syntaxin, Sso, whereas we found two clearly diverged Sso sequences in all other Pezizomycotina, suggesting that this trait emerged after the split of the Eurotiomycetidae. Within the Saccharomycotina, two major clusters were observed, one being the *Candida *clade, which comprises the organisms that translate the codon CTG as serine instead of leucine [87,88] and the other being the *Saccharomyces *complex mentioned above. Generally, these two clusters are clearly separated in all individual trees built from orthologous SNAREs, whereas *Yarrowia lipolytica*, as the sole sequenced member of a third cluster of the Saccharomycotina, splits earlier. Furthermore, our analysis sheds some initial light on the evolutionary position of the few basal fungi available. The concatenated tree includes the two Mucoromycotina, *Rhizopus oryzae *and *Phycomyces blakesleeanus*, and the chytrid fungus *Batrachochytrium dendrobatidis*, all of which are clearly separated from Basidiomycotina.

## Conclusion

Our study confirms that fungi, in contrast to animals and green plants [19,21,34,35,40], generally possess a relatively simple set of SNARE proteins with only minimal variation between different lineages. In addition, our analysis reveals that single-cell yeasts, by and large, did not reduce their primordial set of SNARE proteins, suggesting that they have maintained the basic transport pathways of the proto-eukaryotic cell. In fact, one can observe quite the contrary: for example, the hemiascomycete *S. cerevisiae *has gained a few additional SNAREs during evolution. Taken together, these points imply that a basic set of membrane fusion proteins is able to sustain and is necessary for the lifestyle of multicellular fungi as well as of single-cell yeasts. Hence, multicellularity *per se *does not need an expansion of the repertoire of SNARE proteins.

Moreover, the SNAREs of hemiascomycetes are often more deviated than the ones of basal fungi. These facts highlight that the trafficking pathways of baker's yeast are not only different to those in animal cells but also are different to those of many other fungi. Since the extent of the differences is unknown to date, these ought to be investigated in more detail by comparing the trafficking pathways of yeast with those of more basal fungi.

It is well established that clear differences can be seen in the appearance of organelles of the endocytic system of yeast and animal cells, most strikingly between the yeast vacuole and the animal lysosomes. Yet, is challenging to compare their endocytic systems as both consist of an interconnected and complex network of organelles. The current view is that the endosomal system in yeast is simpler than that in an animal cell, but that they also have common principles and pathways [53,89]. This view is now corroborated by our inspection of the evolution of the SNARE repertoire in fungi. Originally, the endocytic systems of the progenitors of fungal and animal cells probably operated with the same basic SNARE collection inherited from the proto-eukaryotic cell. However, all fungi possess a novel, fungi-specific SNARE factor, Vam7, which can bind to PI(3)P-containing membranes by means of its N-terminal PX-domain. Although the exact function of this SNARE protein is not clear at the moment, it seems to play a role in endosomal trafficking, revealing a clear difference in the endosomal SNARE collection of fungi and animals.

During evolution, the lineage that gave rise to baker's yeast has gained few additional SNARE proteins. The largest transformation of the SNARE collection of baker's yeast was caused by a relatively recent whole genome duplication within the *Saccharomyces *lineage, resulting in an expansion of the secretory set of SNAREs. Although the consequences of this duplication are not well understood, it seems that at least one of the new secretory SNAREs, Spo20, has specialized for the process of sporulation. Another gene duplication took place in the endosomal syntaxins. Earlier in evolution, an ancestral endosomal syntaxin (Qa.III.b) was duplicated, giving rise to Pep12 and Vam3 in the lineage of Saccharomycotina, while other fungi only possess one endosomal syntaxin. It is unclear whether one of the duplicated endosomal syntaxins in hemiascomycetous yeasts retained the ancestral function while the second gained a new function (neofunctionalization) or whether each one carries out part of the ancestral function (subfunctionalization). It seems possible that one of the two syntaxins specialized to the process of homotypic vacuole fusion. This hypothesis could be tested by investigating the function of the ancestral endosomal syntaxin in endosomal trafficking of more basal fungal species like *Ustilago maydis *[90]. The presence of two diverged endosomal syntaxins in hemiascomycetes yeast has also to be taken into consideration when comparing endosomal trafficking in yeast with that in animal cells. In addition, one has to bear in mind that independent and more substantial expansions of the SNARE repertoire occurred in animals, possibly establishing more complex endosomal trafficking pathways [22]. For example, during the evolution of animals, an ancestral endosomal syntaxin (Qa.III.b) gave rise to three different syntaxins, Syx7, Syx20 and Syx17. In addition, several other novel endosomal SNAREs were added. When comparing yeast with animal cells, it is also important not to combine different animal species, as the original animal SNARE collection was modified in many different ways in different animal lineages. For instance, vertebrates possess few additional endosomal SNAREs, e.g. Syx13, although the rise of vertebrates mainly resulted in a robust expansion of the secretory set of SNAREs.

## Abbreviations

AU: Almost Unbiased; ER: endoplasmatic reticulum; EST: expressed sequence tags; FRET: Fluorescence resonance energy transfer; GA: Golgi apparatus; HMM: Hidden Markov Model; IQPNNI: Important Quartet Puzzling and Nearest Neighbor Interchange; JTT: Jones: Taylor and Thornton; Lgl: Lethal giant larvae; NR: non redundant; Npsn: New plant SNARE; NSF: N-ethylmaleimide-sensitive factor; PX: Phox homology; SNARE: soluble NSF Attachment Receptor; TGN: trans Golgi network; WGD: whole genome duplication.

## Authors' contributions

Together the three authors curated the SNARE data base. NK performed the sequence and phylogenetic analysis. THK participated in the phylogenetic analyis and helped to draft the manuscript. THK and DF conceived the study and participated in its design and coordination. DF drafted the manuscript. All authors read and approved the final manuscript.

## Supplementary Material

Additional file 1 **Classification of the complete set of SNARE proteins of *Saccharomyces cerevisiae *according to our HMM models.** The different types are listed according to their intracellular distribution and are allocated into interacting SNARE units based on biological knowledge. In addition, the UniProtKB/Swiss-Prot accession number and the initial descriptions are given for each SNARE protein. Click here for file

Additional file 2 **List of fungal SNAREs. List of SNARE repertoires from different fungi with complete genomes sequenced.** Entire sequences were used to calculate phylogenetic trees. Click here for file

Additional file 3 **Alignment and tree files.** Detailed information to all sequences and trees are contained in a zipped file, which contains all phylogenetic trees in Nexus-format (.nex) and all alignments used to calculate the trees in Fasta-format (.fasta). In addition, a text file is provided ("id2information.txt") that allows to identify the sequences used for our analysis in the NCBI data base: listed is the sequence number & name used in our alignments & trees and the respective gi string. Click here for file

Additional file 4 **Structure-based sequence alignments.** Structure-based sequence alignments of several fungi SNAREs. Click here for file

Additional file 5 **List of SNAREs used as outgroups.** List of SNAREs from species used as outgroups for calculating phylogenetic trees based on sequence alignments of the SNARE motif region only. Click here for file

Additional file 6 **Additional sequence peculiarities of fungal SNAREs.** A text describing few additional sequence peculiarities, in particular of layer residues, of fungal SNAREs. Click here for file
