# Supplementary material for: Phylogeny of the SNARE vesicle fusion machinery yields insights into the conservation of the secretory pathway in fungi
Source: BMC Evol Biol. 2009 Jan 23;9:19. doi: 10.1186/1471-2148-9-19 (PMC2639358; doi:10.1186/1471-2148-9-19)
Supplement: Additional file 1 — Classification of the complete set of SNARE proteins of Saccharomyces cerevisiae according to our HMM models. The different types are listed according to their intracellular distribution and are allocated into interacting SNARE units based on biological knowledge. In addition, the UniProtKB/Swiss-Prot accession number and the initial descriptions are given for each SNARE protein. [file 1471-2148-9-19-S1.pdf]

	Qa-group	Qb-group	Qc-group	R-group
**- I -**<br>**Endoplasmic reticulum**	**Qa.I**<br>*Ufe1 (P41834)*<br>[1]	**Qb.I**<br>*Sec20 (P28791)*<br>[2, 3]	**Qc.I**<br>*Use1 (P53146)*<br>[4-6]	**R.I**<br>*Sec22 (P22214)*<br>[7, 8]
**- II -**<br>**Golgi apparatus**<br>ER-Golgi	**Qa.II**<br>*Sed5 (Q01590)*<br>[9]	**Qb.II**<br>*Bos1 (P25385)*<br>[7, 10]	**Qc.II**<br>*Bet1 (P22804)*<br>[7, 8]	**R.II**<br>*Ykt6 (P36015)*<br>[11]
intra-Golgi		*Gos1 (P38736)*<br>[12]	*Sft1 (P43682)*<br>[13]	
**- III.a -**<br>**Trans Golgi Network**	**Qa.III.a**<br>*Tlg2 (Q08144)*<br>[14, 15]			
**- III.b -**<br>**endosomal compartments**<br>‘early’	**Qa.III.b**<br>*Pep12 (P32854)*<br>[16]/<br>Vam3 *(Q12241)*<br>[17-22]	**Qb.III.b**<br>*Vti1 (Q04338)*<br>[23, 24]	**Qc.III.b**<br>*Tlg1 (Q03322)*<br>[15]	**R.III**<br>*Nyv1 (Q12255)*<br>[22]
‘late’<br>(vacuoles)			**Qc.III.c**<br>*Syx8 (P31377)*<br>[25]<br>/Vam7 *(P32912)*<br>[26]	
**- IV -**<br>**Secretion**<br>(+ Cytokinesis/ Sporulation)	**Qa.IV**<br>*Sso1 (P32867)/*<br>*Sso2 (P39926)*<br>[27]	**SNAP - Qbc.IV**<br>*Sec9 (P40357)* [28]/<br>*Spo20 (Q04359)*<br>[29]		**R.IV**<br>*Snc1 (P31109)/*<br>*Snc2 (P33328)*<br>[30, 31]
**Regulatory**				**R.Reg**<br>*Sro7 (Q12038)/*<br>*Sro77 (P38163)*<br>[32]

Classification of the complete set of SNARE proteins of *Saccharomyces cerevisiae* acording to our HMM models [33].

The different types are listed according to their intracellular distribution and are allocated into interacting SNARE units based on biological knowledge. In addition, the UniProtKB/Swiss-Prot accession number and the initial descriptions are given for each SNARE protein.

References:

1. Lewis MJ, Rayner JC, Pelham HR: **A novel SNARE complex implicated in vesicle fusion with the endoplasmic reticulum**. *Embo J* 1997, **16**(11):3017-3024.
2. Sweet DJ, Pelham HR: **The Saccharomyces cerevisiae SEC20 gene encodes a membrane glycoprotein which is sorted by the HDEL retrieval system**. *Embo J* 1992, **11**(2):423-432.
3. Lewis MJ, Pelham HR: **SNARE-mediated retrograde traffic from the Golgi complex to the endoplasmic reticulum**. *Cell* 1996, **85**(2):205-215.
4. Burri L, Varlamov O, Doege CA, Hofmann K, Beilharz T, Rothman JE, Sollner TH, Lithgow T: **A SNARE required for retrograde transport to the endoplasmic reticulum**. *Proceedings of the National Academy of Sciences of the United States of America* 2003, **100**(17):9873-9877.
5. Dilcher M, Veith B, Chidambaram S, Hartmann E, Schmitt HD, Fischer von Mollard G: **Use1p is a yeast SNARE protein required for retrograde traffic to the ER**. *Embo J* 2003, **22**(14):3664-3674.
6. Belgareh-Touze N, Corral-Debrinski M, Launhardt H, Galan JM, Munder T, Le Panse S, Haguenauer-Tsapis R: **Yeast functional analysis: identification of two essential genes involved in ER to Golgi trafficking**. *Traffic (Copenhagen, Denmark)* 2003, **4**(9):607-617.
7. Newman AP, Shim J, Ferro-Novick S: **BET1, BOS1, and SEC22 are members of a group of interacting yeast genes required for transport from the endoplasmic reticulum to the Golgi complex**. *Mol Cell Biol* 1990, **10**(7):3405-3414.
8. Dascher C, Ossig R, Gallwitz D, Schmitt HD: **Identification and structure of four yeast genes (SLY) that are able to suppress the functional loss of YPT1, a member of the RAS superfamily**. *Mol Cell Biol* 1991, **11**(2):872-885.
9. Hardwick KG, Pelham HR: **SED5 encodes a 39-kD integral membrane protein required for vesicular transport between the ER and the Golgi complex**. *J Cell Biol* 1992, **119**(3):513-521.
10. Shim J, Newman AP, Ferro-Novick S: **The BOS1 gene encodes an essential 27-kD putative membrane protein that is required for vesicular transport from the ER to the Golgi complex in yeast**. *J Cell Biol* 1991, **113**(1):55-64.
11. Sogaard M, Tani K, Ye RR, Geromanos S, Tempst P, Kirchhausen T, Rothman JE, Sollner T: **A rab protein is required for the assembly of SNARE complexes in the docking of transport vesicles**. *Cell* 1994, **78**(6):937-948.
12. McNew JA, Coe JG, Sogaard M, Zemelman BV, Wimmer C, Hong W, Sollner TH: **Gos1p, a Saccharomyces cerevisiae SNARE protein involved in Golgi transport**. *FEBS letters* 1998, **435**(1):89-95.
13. Banfield DK, Lewis MJ, Pelham HR: **A SNARE-like protein required for traffic through the Golgi complex**. *Nature* 1995, **375**(6534):806-809.
14. Abeliovich H, Grote E, Novick P, Ferro-Novick S: **Tlg2p, a yeast syntaxin homolog that resides on the Golgi and endocytic structures**. *The Journal of biological chemistry* 1998, **273**(19):11719-11727.
15. Holthuis JC, Nichols BJ, Dhruvakumar S, Pelham HR: **Two syntaxin homologues in the TGN/endosomal system of yeast**. *Embo J* 1998, **17**(1):113-126.
16. Jones EW: **Proteinase mutants of Saccharomyces cerevisiae**. *Genetics* 1977, **85**(1):23-33.
17. Wada Y, Ohsumi Y, Anraku Y: **Genes for directing vacuolar morphogenesis in Saccharomyces cerevisiae. I. Isolation and characterization of two classes of vam mutants**. *The Journal of biological chemistry* 1992, **267**(26):18665-18670.
18. Wada Y, Nakamura N, Ohsumi Y, Hirata A: **Vam3p, a new member of syntaxin related protein, is required for vacuolar assembly in the yeast Saccharomyces cerevisiae**. *J Cell Sci* 1997, **110**(Pt 11):1299-1306.
19. Götte M, Gallwitz D: **High expression of the yeast syntaxin-related Vam3 protein suppresses the protein transport defects of a pep12 null mutant**. *FEBS letters* 1997, **411**(1):48-52.

20. Darsow T, Rieder SE, Emr SD: **A multispecificity syntaxin homologue, Vam3p, essential for autophagic and biosynthetic protein transport to the vacuole**. *J Cell Biol* 1997, **138**(3):517-529.
21. Sato TK, Darsow T, Emr SD: **Vam7p, a SNAP-25-like molecule, and Vam3p, a syntaxin homolog, function together in yeast vacuolar protein trafficking**. *Mol Cell Biol* 1998, **18**(9):5308-5319.
22. Nichols BJ, Ungermann C, Pelham HR, Wickner WT, Haas A: **Homotypic vacuolar fusion mediated by t- and v-SNAREs [see comments]**. *Nature* 1997, **387**(6629):199-202.
23. Fischer von Mollard G, Nothwehr SF, Stevens TH: **The yeast v-SNARE Vti1p mediates two vesicle transport pathways through interactions with the t-SNAREs Sed5p and Pep12p**. *J Cell Biol* 1997, **137**(7):1511-1524.
24. Lupashin VV, Pokrovskaya ID, McNew JA, Waters MG: **Characterization of a Novel Yeast SNARE Protein Implicated in Golgi Retrograde Traffic**. *Molecular biology of the cell* 1997, **8**(12):2659-2676.
25. Lewis MJ, Pelham HR: **A new yeast endosomal SNARE related to mammalian syntaxin 8**. *Traffic (Copenhagen, Denmark)* 2002, **3**(12):922-929.
26. Wada Y, Anraku Y: **Genes for directing vacuolar morphogenesis in Saccharomyces cerevisiae. II. VAM7, a gene for regulating morphogenic assembly of the vacuoles**. *The Journal of biological chemistry* 1992, **267**(26):18671-18675.
27. Aalto MK, Ronne H, Keranen S: **Yeast syntaxins Sso1p and Sso2p belong to a family of related membrane proteins that function in vesicular transport**. *Embo J* 1993, **12**(11):4095-4104.
28. Brennwald P, Kearns B, Champion K, Keranen S, Bankaitis V, Novick P: **Sec9 is a SNAP-25-like component of a yeast SNARE complex that may be the effector of Sec4 function in exocytosis**. *Cell* 1994, **79**:245-258.
29. Neiman AM: **Prospore membrane formation defines a developmentally regulated branch of the secretory pathway in yeast**. *J Cell Biol* 1998, **140**(1):29-37.
30. Gerst JE, Rodgers L, Riggs M, Wigler M: **SNC1, a yeast homolog of the synaptic vesicle-associated membrane protein/synaptobrevin gene family: genetic interactions with the RAS and CAP genes**. *Proceedings of the National Academy of Sciences of the United States of America* 1992, **89**(10):4338-4342.
31. Protopopov V, Govindan B, Novick P, Gerst JE: **Homologs of the synaptobrevin/VAMP family of synaptic vesicle proteins function on the late secretory pathway in S. cerevisiae**. *Cell* 1993, **74**(5):855-861.
32. Kagami M, Toh-e A, Matsui Y: **Sro7p, a Saccharomyces cerevisiae counterpart of the tumor suppressor l(2)gl protein, is related to myosins in function**. *Genetics* 1998, **149**(4):1717-1727.
33. Kloepper TH, Kienle CN, Fasshauer D: **An elaborate classification of SNARE proteins sheds light on the conservation of the eukaryotic endomembrane system**. *Molecular biology of the cell* 2007, **18**(9):3463-3471.
