# Supplementary material for: Phylogeny of the SNARE vesicle fusion machinery yields insights into the conservation of the secretory pathway in fungi
Source: BMC Evol Biol. 2009 Jan 23;9:19. doi: 10.1186/1471-2148-9-19 (PMC2639358; doi:10.1186/1471-2148-9-19)
Supplement: Additional file 2 — List of fungal SNAREs. List of SNARE repertoires from different fungi with complete genomes sequenced. Entire sequences were used to calculate phylogenetic trees. [file 1471-2148-9-19-S2.pdf]

Species Groups				Species	Endoplasmatic Reticulum				Golgi Aparatus						TGN	Endosomes / Lysosomes								Secretion			Polarized
					Qa.I	Qb.I	Qc.I	R.I	Qa.II	Qb.II		Qc.II		R.II	Qa.III.a	Qa.III.b		Qb.III.b	Qb.III.d	Qc.III			R.III	Qa.IV	Qbc.IV	R.IV	R.Reg
					Ufe1	Sec20	Use1	Sec22	Sed5	Bos1	Gos1	Bet1	Sft1	Ykt6	Tlg2	Pep12	Vam3	Vti1	Qb.III.d/Npsn	Tlg1	Vam7	Syx8	Nyv1	Sso	Sec9	Snc	Tomosyn
**Basal Fungi**	**Blastocladiomycotina**			Cunninghamella elegans				•			•				•			•					•	•	•	•	
				Allomyces macrogynus										•											•	••	
				Blastocladiella emersonii				•		•	•	•		•				•	•	••		•		•	•	•••	
	**Mucoromycotina**			Rhizopus oryzae	•	•	•	•	•	•			•	••	•	•		•		•	•	•	•	•••	•	•	•
				Phycomyces blakesleeanus	•	•	•	•	•	•	•		•	•	•	•		•		•	•	•	•	••••		••	•
	**Chytridiomycota**			Batrachochytrium dendrobatidis	•			•	•	•	•	•	•	•	•	•		•	•	•	•	•	•	••	•	•	•
**Basidiomycota**	**Ustilagomycotina**			Ustilago maydis	•	•	•	•	•	•	•	•	•	•	•	•		•		•	•	•	•	•	•	•	•
				Malassezia globosa	•	•	•	•	•	•	•			•	•	•		•		•	•	•		•	•	•	•
	**Pucciniomycotina**			Sporobolomyces roseus	•		•	•	•		•	•	•	•	•	•		•		•	•	•	•	••	•	•	
				Puccinia graminis	•	•		•	•		•	•	•	•	•	•		•			•	•	•	•	•	•	•
				Phakopsora pachyrhizi			•			•	•	•		•	•					•	•			••	•		
	**Agaricomycotina**			Cryptococcus neoformans	•	•	•	•	•	•	•	•	•	•	•	•		•		•	•	•	•	•	•	•	•
				Coprinus cinereus	•	•	•	•	•	•	•		•	•	•	•		•		•	•	•	•	•	•	•	•
				Laccaria bicolor	•	•	•	•	•	•	•	•	•	•	•	•		•		•	•	•	•	•	•	•	•
				Phanerochaete chrysosporium	•	•	•	•	•	•	•	•	•	•	•	•		•		•	•	•	•	••	•	•	
				Postia placenta	•	•	•	•	•	•	•	•	•	•	•	•		•		•	•	•	•	••	•	•	•
**Ascomycota**	**Schizosaccharomycetes**			Schizosaccharomyces pombe	•	•	•	•	•	•	•	•	•	•	•	•		•		•	•			•	•	•	•
				Schizosaccharomyces japonicus	•	•	•	•	•	•	•	•	•	•	•	•		•		•	•			•	•	•	•
	**Pezizomycotina**	**Sodariomycetes**		Neurospora crassa	•	•	•	•	•	•	•	•	•	•	•	•		•		•	•	•	•	••	•	•	•
				Gibberella zeae	•	•	•	•	•	•	•	•	•	•	•	•		•		•	•	•	•	••	•	•	•
				Podospora anserina	•	•	•	•	•	•	•	•	•	•	•	•		•		•	•	•	•	••	•	•	•
				Magnaporthe grisea	•	•	•	•	•	•	•	•	•	•	•	•		•		•	•	•	•	••	•	•	•
				Fusarium verticillioides	•	•	•	•	•	•	•	•	•	•	•	•		••		•	•	•	•	••	•	•	•
				Nectria haematococca	•	•	•	•	•	•	•	•	•	•	•	•		•		•	•	•	•	••	•	•	•
				Chaetomium globosum	•		•	•	•	•	•	•	•	•	•	•		•		•	•	•	••	••	•	•	•
				Trichoderma reesei	•	•	•	•	•	•	•	•	•	•	•	•		•		•	•	•	•	••	•	•	•
				Fusarium oxysporum	•	•	•	•	•	•	•	•	•	•	•	•		•		•	•	•	•	••	•	•	•
				Trichoderma virens	•	•	•	•	•	•	•	•	•	•	•	•		•		•	•	•	•	••	•	•	•
				Trichoderma atroviride	•		•	•	•	•	•	•	•	•	•	•		•		•	•	•	•	•	•	•	•
		**Leotiomycetes**		Sclerotinia sclerotiorum	•	•	•	•	•	•	•	•	•	•	•	•		•		•	•	•	•	••	•	•	•
				Botrytis cinerea		•		•	•	•	•	•	•	•	•			•		•	•	•	•	••	•	•	•
		**Dothideomycetes**		Mycosphaerella graminicola	•	•	•	•	•	•	•	•	•	•	•	•		•		•	•	•	•	••	•	•	•
				Phaeosphaeria nodorum	•	•	•	•	•	•	•	•	•	•	•	•		•		•	•	•	•	••	•	•	•
				Mycosphaerella fijiensis	•	•	•	•	•	•	•	•	•	•				•		•	•	•	•	••	•	•	•
				Cochliobolus heterostrophus	•	•	•	•	•	•		•	•	•	•	•		•		•	•	•	•	•••	•	•	•
				Pyrenophora triticirepentis	•	•	•	•	•	•	•	•		•		•		•		•	•	•	•	•	•	•	•
		**Eurotiomycetes**	**Onygenales**	Histoplasma capsulatum	•	•		•	•	•	•	•	•	•	•	•		•		•	•	•	•	•	•	•	
				Uncinocarpus reesii	•	•	•	•	•	•	•	•	•	•	•	•		•		•	•	•	•	•	•	•	•
				Coccidioides immitis	•	•	•	•	•	•	•	•		•	•	•		•		•	•	•	•	•	•	•	•
				Coccidioides posadasii	•	•	•	•	•	•	•	•	•	•	•	•		•		•	•	•	•	•	•	•	•
				Paracoccidioides brasiliensis	•	•	•	•	•	•	•	•		•		•		•		•	•	•	•	•	•	•	•
			**Eurotiales**	Aspergillus nidulans	•	•	•	•	•		•	•	•	•	•	•		•		•	•	•	•	•	•	•	•
				Aspergillus niger	•	•	•	•	•	•	•	•	•	•	•	•		•		•	•	•	•	•	•	•	•
				Aspergillus oryzae	•	•	•	•	•	•	•	•	•	•	•	•		•		•	•	•	•	••	•	•	•
				Aspergillus fumigatus	•	•	•	•	•	•	•	•	•	•	•	•		•		•	•	•	•	•	•	•	•
				Aspergillus terreus	•	•	•	•	•	•	•	•	•	•	•	•		•		•	•	•	•	•	•	•	•
				Aspergillus clavatus	•	•	•	•	•	•	•	•	•	•	•	•		•		•	•	•	•	•	•	•	•
				Neosartorya fischeri	•	•	•	•	•	•	•	•	•	•	•	•		•		•	•	•	•	•	•	•	•
				Aspergillus flavus	•	•	•	•	•	•	•	•	•	•	•	•		•		•	•	•	•	••	•	•	•
	**Saccharomycotina**			Yarrowia lipolytica	•	•	•	•	•	•	•	•	•	•	•	•	•	•		•	•	•	•	•••	•	••	•
		**Candida clade**		Debaryomyces hansenii	•	•	•	•	•	•	•	•		•	•	•	•	•		•	•	•	•	••	•	•	•
				Candida albicans	•	•	•	•	•	•	•	•	•	•	•	•	•	•		•	•	•	•	•	•	•	•
				Candida guilliermondii	•	•	•	•	•	•	•	•	•	•	•	•	•	•		•	•	•	•	•	•	•	•
				Candida lusitaniae	•	•	•	•	•	•	•	•	•	•	•	•	•	•		•	•	•	•	•	•	•	•
				Pichia stipitis	•	•	•	•	•	•	•	•	•	•	•	•	•	•		•	•	•	•	••	•	•	
				Candida tropicalis	•	•	•	•	•	•	•	•	•	•	•	•	•	•		•	•	•	•	•	•	•	•
				Lodderomyces elongisporus	•	•	•	•	•	•	•	•	•	•	•	•	•	•		•	•	•	•	•	•	•	•
				Candida dubliniensis	•	•	•	•	•	•	•	•	•	•		•	•	•		•	•	•	•	•	•	•	•
				Candida parapsilosis	•	•	•	•	•	•	•	•	•	•	•	•	•	•		•	•	•	•	•	•		•
		**Saccharomyces clade**		Eremothecium gossypii	•	•	•	•	•	•	•	•	•	•	•	•	•	•		•	•	•	•	•	•	•	•
				Saccharomyces cerevisiae	•	•	•	•	•	•	•	•	•	•	•	•	•	•		•	•	•	•	••	••	••	••
				Kluyveromyces lactis	•	•	•	•	•	•	•	•	•	•	•	•	•	•		•	•	•	•	•	•	•	•
				Candida glabrata	•	•	•	•	•	•	•	•		•	•	•	•	•		•	•	•	•	••	•	••	
				Saccharomyces paradoxus	•	•	•	•	•	•	•	•	•	•	•	•	•	•		•	•	•	•	••	••	••	••
				Saccharomyces mikatae	•	•	•	•	•	•	•	•	•	•	•	•	•	•		•	•	•	•	••	••	••	•
				Saccharomyces bayanus	•	•	•	•	•	•	•	•	•	•	•	•	•	•		•	•	•	•	••	••	••	••
				Saccharomyces kudriavzevii	•	•	•	•	•	•	•	•	•		•	•	•	•		•	•			••	••	••	•
				Saccharomyces castellii	•	•	•	•	•	•	•	•		•	•	•	•	•		•		•	•	••	••	•	•
				Saccharomyces kluyveri	•	•	•	•	•	•	•	•	•	•	•	•	•	•		•	•	•	•	•	•	•	•
				Kluyveromyces waltii	•	•	•	•	•	•	•		•	••	•	•	•	•		•	•	•	•	•	•	•	•
				Vanderwaltozyma polyspora	•	•	•	•	•	•	•	•	•		•	•	•	•		•	•	•	•	•	••	•	
