# Supplementary material for: Phylogeny of the SNARE vesicle fusion machinery yields insights into the conservation of the secretory pathway in fungi
Source: BMC Evol Biol. 2009 Jan 23;9:19. doi: 10.1186/1471-2148-9-19 (PMC2639358; doi:10.1186/1471-2148-9-19)
Supplement: Additional file 5 — List of SNAREs used as outgroups. List of SNAREs from species used as outgroups for calculating phylogenetic trees based on sequence alignments of the SNARE motif region only. [file 1471-2148-9-19-S5.pdf]

Species	Endoplasmatic Reticulum					Golgi Aparatus								TGN	Endosomes / Lysosomes														Secretion						Polarized		
	Qa.I	Qb.I	Qc.I	R.I		Qa.II	Qb.II				Qc.II		R.II	Qa.III.a	Qa.III.b					Qb.III.b	Qb.III.d	Qc.III.b	Qc.III.c			R.III			Qa.IV	Qbc.IV			R.IV		R.Reg		
	Syx18	Sec20	Use1	Sec22	Sec22like	Syx5	Bos1	Gos1	Memb	Gos28	Bet1	Gs15	Ykt6	Syx16	Syx7	Syx13	Pep12	Syx17	Syx20	Vti1	Qb.III.d/Npsn	Syx6	Syx8	Syp7	Syx6like	Vamp7	Vamp7-like/Endobrevin	Vamp4	Syx1	SN25	SN29	SN47	Syb	Myob	Tomosyn	Ami	Lgl
**Encephalitozoon cuniculi**		▪	▪				▪						▪	▪			▪									▪											
**Capsaspora owczarzaki**							▪	▪												▪						▪			▪				▪				
**Sphaeroforma arctica**															▪											▪							▪▪				
**Monosiga ovata**						▪							▪	▪	▪▪					▪		▪	▪▪			▪			▪▪	▪▪			▪▪				
**Monosiga brevicollis**	▪			▪		▪			▪	▪		▪	▪	▪	▪					▪▪		▪	▪			▪▪			▪	▪			▪		▪		
**Trichoplax adhaerens**	▪	▪	▪	▪		▪			▪	▪		▪	▪	▪	▪			▪	▪	▪▪		▪	▪			▪▪	▪		▪▪▪▪	▪▪		▪	▪▪		▪		▪
**Dictyostelium discoideum**	▪	▪	▪	▪		▪	▪	▪			▪		▪▪	▪	▪▪					▪	▪▪▪		▪▪	▪	▪▪	▪			▪▪▪	▪			▪▪		▪		
**Helobdella robusta**	▪	▪	▪	▪		▪			▪	▪	▪	▪	▪▪	▪	▪				▪	▪		▪				▪▪			▪▪▪	▪▪	▪		▪▪▪		▪▪		▪
**Lottia gigantea**	▪	▪	▪	▪	▪	▪			▪	▪	▪	▪	▪	▪				▪	▪	▪▪		▪	▪			▪	▪	▪▪	▪	▪	▪	▪	▪		▪		▪
**Branchiostoma floridae**	▪	▪	▪	▪	▪	▪			▪	▪	▪	▪	▪	▪	▪			▪	▪	▪▪		▪	▪			▪▪		▪	▪	▪▪	▪	▪	▪		▪▪		▪
**Ciona intestinalis**		▪	▪	▪		▪			▪	▪	▪	▪	▪		▪			▪		▪▪		▪	▪			▪	▪	▪	▪▪▪	▪	▪		▪		▪		▪
**Daphnia pulex**	▪	▪	▪	▪		▪			▪	▪		▪	▪	▪▪	▪			▪	▪	▪▪		▪	▪			▪▪		▪	▪▪▪	▪	▪		▪▪		▪		
**Homo sapiens**	▪	▪	▪	▪	▪▪	▪			▪	▪	▪	▪	▪	▪	▪	▪		▪	▪	▪▪		▪▪	▪			▪	▪	▪	▪▪▪▪▪▪▪	▪▪▪	▪	▪	▪▪▪	▪	▪▪	▪	▪▪
**Xenopus laevis**	▪	▪	▪	▪	▪▪	▪			▪	▪	▪	▪	▪	▪	▪				▪	▪		▪	▪			▪	▪		▪▪▪▪▪	▪▪▪	▪		▪▪			▪	▪▪
