# Supplementary material for: Phylogeny of the SNARE vesicle fusion machinery yields insights into the conservation of the secretory pathway in fungi
Source: BMC Evol Biol. 2009 Jan 23;9:19. doi: 10.1186/1471-2148-9-19 (PMC2639358; doi:10.1186/1471-2148-9-19)
Supplement: Additional file 6 — Additional sequence peculiarities of fungal SNAREs. A text describing few additional sequence peculiarities, in particular of layer residues, of fungal SNAREs. [file 1471-2148-9-19-S6.pdf]

Additional sequence peculiarities of fungal SNAREs

It is worth mentioning that several other SNARE proteins exhibit some sequence peculiarities. In all fungi, besides some sequences which seem to be misassembled, the Qb-SNARE involved in retrograde transport from the GA to the ER, Sec20 (Qb.I) [1], generally holds a long C-terminal extension. Notably, a short stretch of the C-terminal extension that follows the transmembrane region contains several hydrophobic amino acids, suggesting that it also interacts with the membrane. This short stretch is followed by a longer, probably luminal, sequence that terminates in a conserved, functional retrieval signal for the KDEL receptor [2]. The C-terminal retrieval signal is not a molecular invention of fungi as it is present in Sec20 in other eukaryotes, for example in green plants, suggesting that it denotes a archetypical trait of this SNARE. Interestingly, Sec20 in animals has lost the long C-terminal extension with a retrieval signal. Another substantial luminal extension was found in Tlg2 (Qa.III.a; syntaxin 16 in animals), the syntaxin localized to the TGN [3, 4]. This type of C-terminal extension was not found in the homologous factors from animals and plants.

A few Q-SNAREs from baker´s yeast, Bet1 (Qc.II), Sft1 (Qc.II) and Use1 (Qc.I), were found not to possess the usual glutamine residue in the 0-layer position, but to carry the unusual residues serine (Bet1) or aspartate (Sft1 and Use1). In this regard, we would like to point out that in one of the subunits of the SNARE complex involved in early endosome trafficking in animals, Vti1a, the 0-layer glutamine is also substituted by an aspartate. The crystal structure showed that the aspartate occupies the same position as the glutamine in other complexes [5], revealing that other residues can be accommodated in the four-helix bundle complex. Interestingly, the 0-layer position of Bet1 in other fungi is conserved, containing either a serine or a threonine residue. The 0-layer aspartate of Use1 is also highly conserved, yet a similarly charged glutamate residue was found in Eurotiomycetes and some Dothideomycetes. In contrast, the sequence surrounding the 0-layer position in Sft1 is less conserved. We observed that the Basidiomycotina still possess a canonical glutamine residue. A glutamine is also present in several other fungi, particularly in Pezizomycotina. Rather atypically, in these species, an amino acid was lost between the 0-layer and the +1-layer.

Judging solely from their sequences, it is debatable whether the two fungal Qc.II-SNAREs, Bet1 and Sft1, are directly homologous to the two Qc.II-SNAREs in

animals, Bet1 and Gs15, respectively. Yet, along with the Qa.II-SNARE Sed5 (Syx5 in animals), the two Qc.II-SNAREs Bet1 and Sft1 are believed to interact preferentially with Qb.II-SNAREs Bos1 (Membrin in animals) and Gos1 (Gos28 in animals), respectively [6]. Though the data are partly inconsistent, it still appears that this pattern of interaction of GA SNAREs is preserved between fungi and animals [7-11].

While Sft1 in several fungi exhibits a gap in close proximity to the 0-layer, the Qc.III-SNARE Syx8 in a lineage of Saccharomycotina, referred to as the "*Saccharomyces* complex", contains an extra amino acid between 0- and +1-layer. We found different residues at this position, in two species, we even discovered a glycine. It would be interesting to find out how such substantial alterations are affecting the overall integrity and functionality of the four-helix bundle SNARE complex.

As a last point, we would like to mention that the Qb.I-SNARE Sec20 of yeast contains a glutamine residue in the 0-layer. However, a 0-layer glutamine is confined to most Saccharomycotina, whereas most other fungi possess a serine in this position. Notably, a serine can also be found in the 0-layer of Sec20 in animals and other eukaryotic lineages.

References:

1. Lewis MJ, Pelham HR: **SNARE-mediated retrograde traffic from the Golgi complex to the endoplasmic reticulum**. *Cell* 1996, **85**(2):205-215.
2. Sweet DJ, Pelham HR: **The Saccharomyces cerevisiae SEC20 gene encodes a membrane glycoprotein which is sorted by the HDEL retrieval system**. *Embo J* 1992, **11**(2):423-432.
3. Holthuis JC, Nichols BJ, Dhruvakumar S, Pelham HR: **Two syntaxin homologues in the TGN/endosomal system of yeast**. *Embo J* 1998, **17**(1):113-126.
4. Abeliovich H, Grote E, Novick P, Ferro-Novick S: **Tlg2p, a yeast syntaxin homolog that resides on the Golgi and endocytic structures**. *The Journal of biological chemistry* 1998, **273**(19):11719-11727.
5. Zwilling D, Cypionka A, Pohl WH, Fasshauer D, Walla PJ, Wahl MC, Jahn R: **Early endosomal SNAREs form a structurally conserved SNARE complex and fuse liposomes with multiple topologies**. *Embo J* 2007, **26**(1):9-18.
6. Tsui MM, Tai WC, Banfield DK: **Selective formation of sed5p-containing snare complexes is mediated by combinatorial binding interactions**. *Molecular biology of the cell* 2001, **12**(3):521-538.
7. Hay JC, Klumperman J, Oorschot V, Steegmaier M, Kuo CS, Scheller RH: **Localization, dynamics, and protein interactions reveal distinct roles for ER and golgi SNAREs**. *J Cell Biol* 1998, **141**(7):1489-1502.
8. Parlati F, Varlamov O, Paz K, McNew JA, Hurtado D, Sollner TH, Rothman JE: **Distinct SNARE complexes mediating membrane fusion in Golgi transport based on combinatorial specificity**. *Proceedings of the National Academy of Sciences of the United States of America* 2002, **99**(8):5424-5429.
9. Xu Y, Martin S, James DE, Hong W: **GS15 forms a SNARE complex with syntaxin 5, GS28, and Ykt6 and is implicated in traffic in the early cisternae of the Golgi apparatus**. *Molecular biology of the cell* 2002, **13**(10):3493-3507.
10. Shorter J, Beard MB, Seemann J, Dirac-Svejstrup AB, Warren G: **Sequential tethering of Golgins and catalysis of SNAREpin assembly by the vesicle-tethering protein p115**. *J Cell Biol* 2002, **157**(1):45-62.
11. Tai G, Lu L, Wang TL, Tang BL, Goud B, Johannes L, Hong W: **Participation of the syntaxin 5/Ykt6/GS28/GS15 SNARE complex in transport from the early/recycling endosome to the trans-Golgi network**. *Molecular biology of the cell* 2004, **15**(9):4011-4022.
