# Supplementary material for: Phylogeny of the SNARE vesicle fusion machinery yields insights into the conservation of the secretory pathway in fungi
Source: BMC Evol Biol. 2009 Jan 23;9:19. doi: 10.1186/1471-2148-9-19 (PMC2639358; doi:10.1186/1471-2148-9-19)
Supplement: Additional file 4 — Structure-based sequence alignments. Structure-based sequence alignments of several fungi SNAREs. [file 1471-2148-9-19-S4.pdf]

Structure-based sequence alignments

For several SNARE proteins from *S. cerevisiae,* high-resolution structures are available. The respective structural information was incorporated into the following sequence alignments. In addition, for a few factors (Sec22 and Vti1a), the structural information from homologous SNAREs from vertebrates were included. The names of the proteins, the SNARE-type and the pdb accession code are listed. Generally, the SNARE motif and the transmembrane region are indicated. The illustrations were generated using JalView [1].

a) Ykt6 (R.II, 1H8M and 1IOU). The N-terminal domain of Ykt6 adopts a profilin-like fold [2].

b) Nyv1 (R.III, 2FZ0). Like Ykt6 and Sec22, the N-terminal longin domain exhibits a profilin-like fold [3]).

c) Sro7 (R.Reg, 2OAJ,) The tomosyn homolog from bakers´yeast has a reduced C-terminal SNARE motif. The crystal structure includes the N-terminal propeller, the C-terminal propeller, the 8AB loop, and a tail loop [4].

d) Vam3 (Qa.III.b, 1HS7). The N-terminal domain of Vam3 folds into a three-helix bundle structure [5].

e) Pep12 (Qa.III.b). For the alignment of Pep12, the structural information of the close homolog of Vam3 was used.

f) Vam7 (Qc.III, 1KMD). The structure of the N-terminal PX domain was solved by NMR [6].

e) Sed5 (Qa.II, 1MQS). The crystal structure of the complex between the SM protein Sly1 with the syntaxin Sed5 includes only the N-terminal portion of Sed5 [7].

f) Sso (Qa.IV, 1FIO). The crystal structure of Sso exhibits a closed conformation, in which the N-terminal three-helix bundle portion (referred to as the Habc-domain) folds back onto the SNARE motif [8]. The SNARE motif (referred to as the H3-domain) was also crystallized in the SNARE complex (3B5N) with Sec9 and Snc [9].

g) Sec9 (Qab.IV, 3B5N, [9]).

h) Snc (R.IV, 3B5N, [9])

i) Tlg1 (Qc.III, 2C5I, 2C5J, and 2C5K). The crystal structure of the N-terminal domain of Tlg1 with a peptide of Vps51 exhibits a three-helix bundle structure found in other Q-SNAREs [10].

j) Bet1 (Qc.II). The COPII coat subunit, Sec24, was crystallized in complex with a peptide from the SNARE protein Bet1. The peptide is located on the N-terminal side of the SNARE motif of Bet1 [11].

k) Sec22 (R.I, 2NUT). The crystal structure of the N-terminal domain of Sec22 found in mice exhibits a profilin-like fold [12].
l) Vti (Qb.III, 1VCS). The N-terminal domain of Vti1a from *Mus musculus* exhibits a three-helix bundle structure.

References:

1. Clamp M, Cuff J, Searle SM, Barton GJ: **The Jalview Java alignment editor**. *Bioinformatics (Oxford, England)* 2004, **20**(3):426-427.
2. Tochio H, Tsui MM, Banfield DK, Zhang M: **An autoinhibitory mechanism for nonsyntaxin SNARE proteins revealed by the structure of Ykt6p**. *Science (New York, NY* 2001, **293**(5530):698-702.
3. Wen W, Chen L, Wu H, Sun X, Zhang M, Banfield DK: **Identification of the yeast R-SNARE Nyv1p as a novel longin domain-containing protein**. *Molecular biology of the cell* 2006, **17**(10):4282-4299.
4. Hattendorf DA, Andreeva A, Gangar A, Brennwald PJ, Weis WI: **Structure of the yeast polarity protein Sro7 reveals a SNARE regulatory mechanism**. *Nature* 2007, **446**(7135):567-571.
5. Dulubova I, Yamaguchi T, Wang Y, Sudhof TC, Rizo J: **Vam3p structure reveals conserved and divergent properties of syntaxins**. *Nat Struct Biol* 2001, **8**(3):258-264.
6. Lu J, Garcia J, Dulubova I, Sudhof TC, Rizo J: **Solution structure of the Vam7p PX domain**. *Biochemistry* 2002, **41**(19):5956-5962.
7. Bracher A, Weissenhorn W: **Structural basis for the Golgi membrane recruitment of Sly1p by Sed5p**. *Embo J* 2002, **21**(22):6114-6124.
8. Munson M, Chen X, Cocina AE, Schultz SM, Hughson FM: **Interactions within the yeast t-SNARE sso1p that control SNARE complex assembly**. *Nature Struct Biol* 2000, **7**(10):894-902.
9. Strop P, Kaiser SE, Vrljic M, Brunger AT: **The structure of the yeast plasma membrane SNARE complex reveals destabilizing water-filled cavities**. *The Journal of biological chemistry* 2008, **283**(2):1113-1119.
10. Fridmann-Sirkis Y, Kent HM, Lewis MJ, Evans PR, Pelham HR: **Structural analysis of the interaction between the SNARE Tlg1 and Vps51**. *Traffic (Copenhagen, Denmark)* 2006, **7**(2):182-190.
11. Mossessova E, Bickford LC, Goldberg J: **SNARE selectivity of the COPII coat**. *Cell* 2003, **114**(4):483-495.
12. Gonzalez LC, Jr., Weis WI, Scheller RH: **A novel SNARE N-terminal domain revealed by the crystal structure of Sec22b**. *The Journal of biological chemistry* 2001, **17**:17.

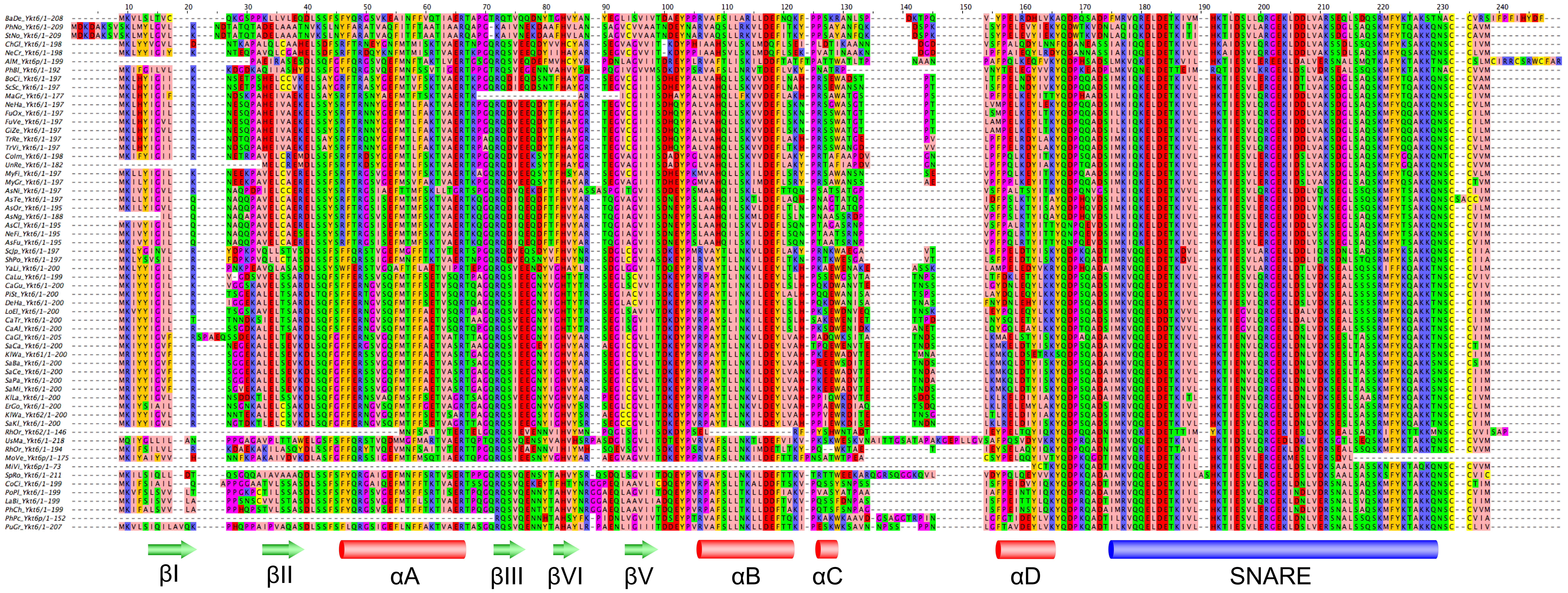
βI
βII
αA
βIII
βVI
βV
αB
αC
αD
SNARE

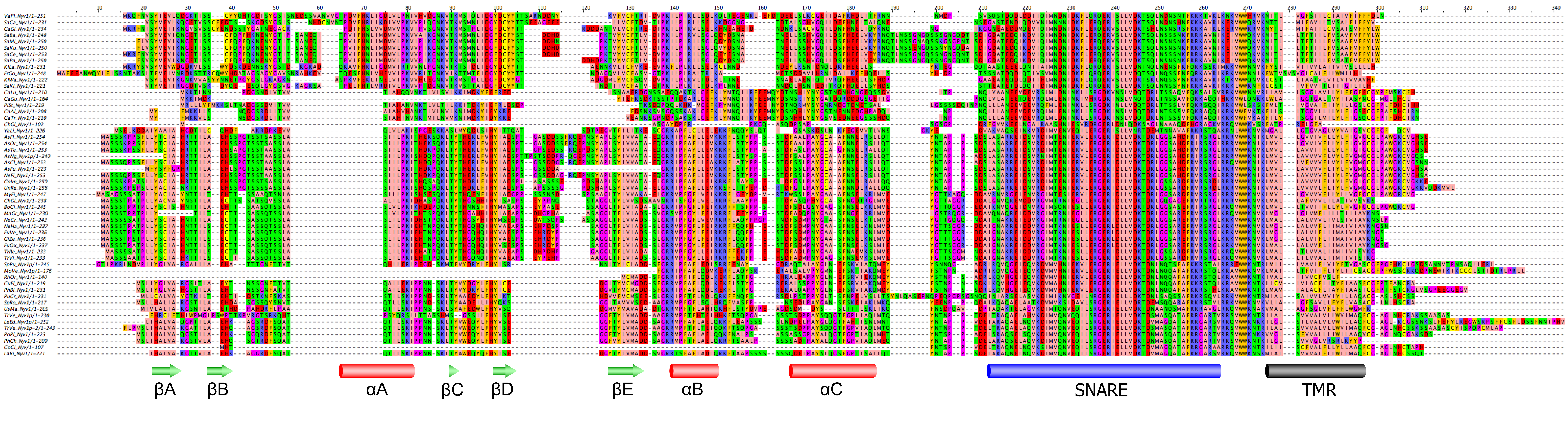
βA
βB
αA
βC
βD
βE
αB
αC
SNARE
TMR

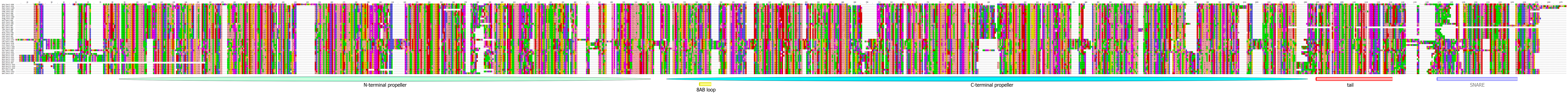
N-terminal propeller
8AB loop
C-terminal propeller
tail
SNARE

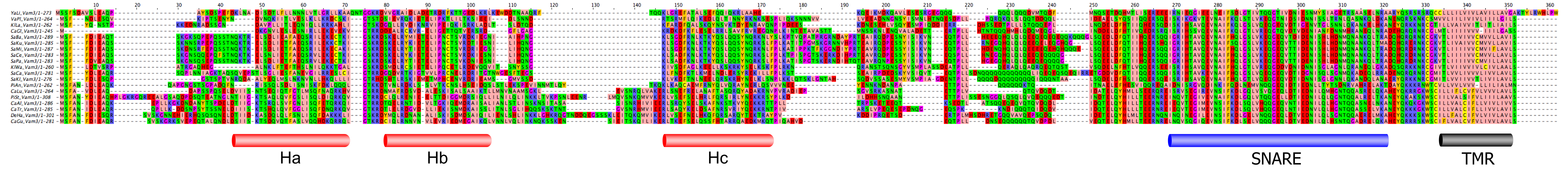
Ha
Hb
Hc
SNARE
TMR

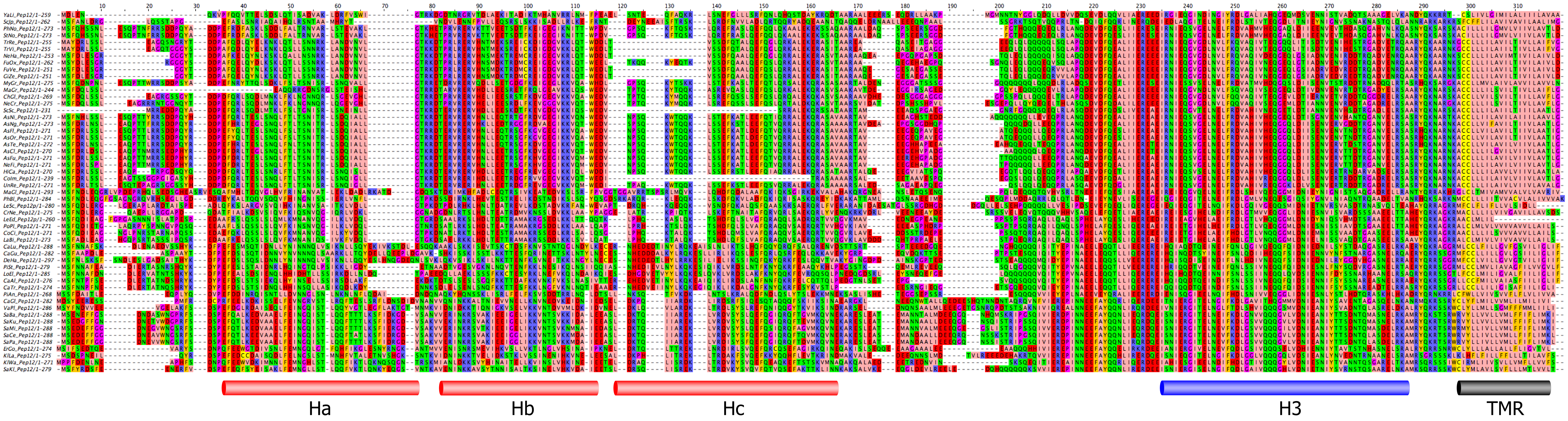
Ha
Hb
Hc
H3
TMR

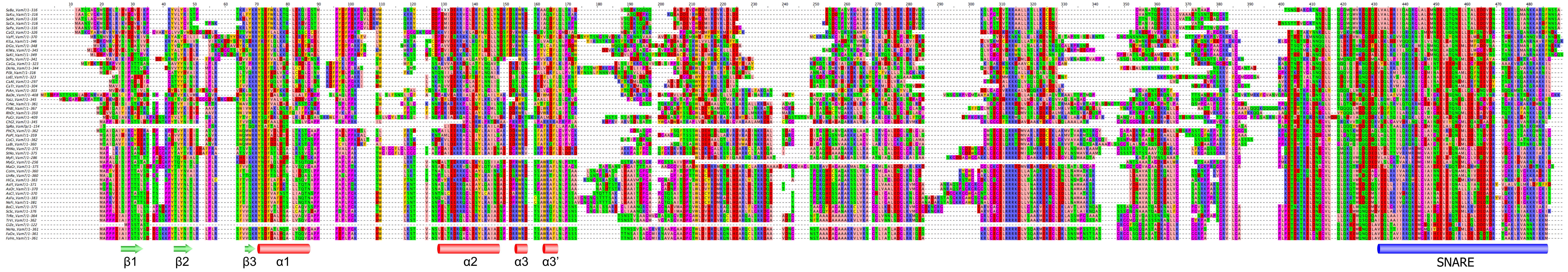
β1
β2
β3
α1
α2
α3
α3'
SNARE

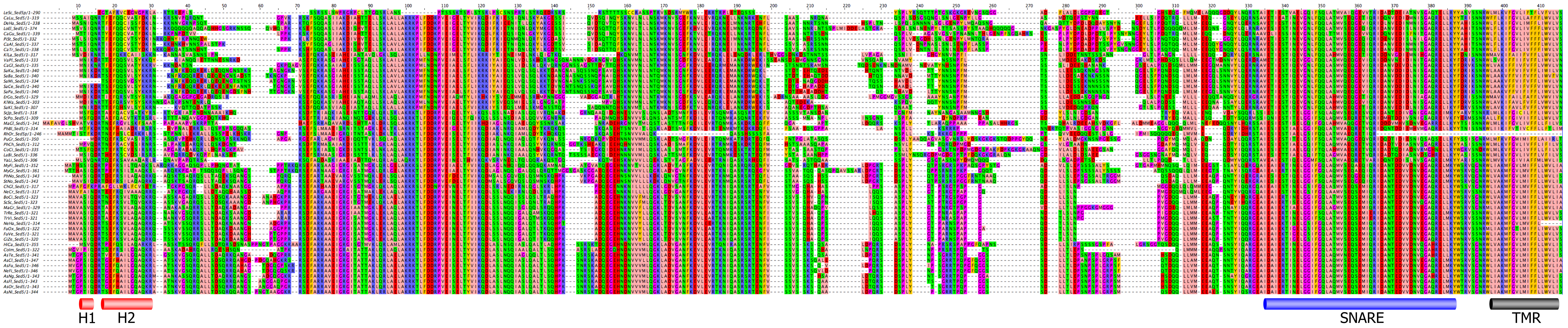
H1
H2
SNARE
TMR

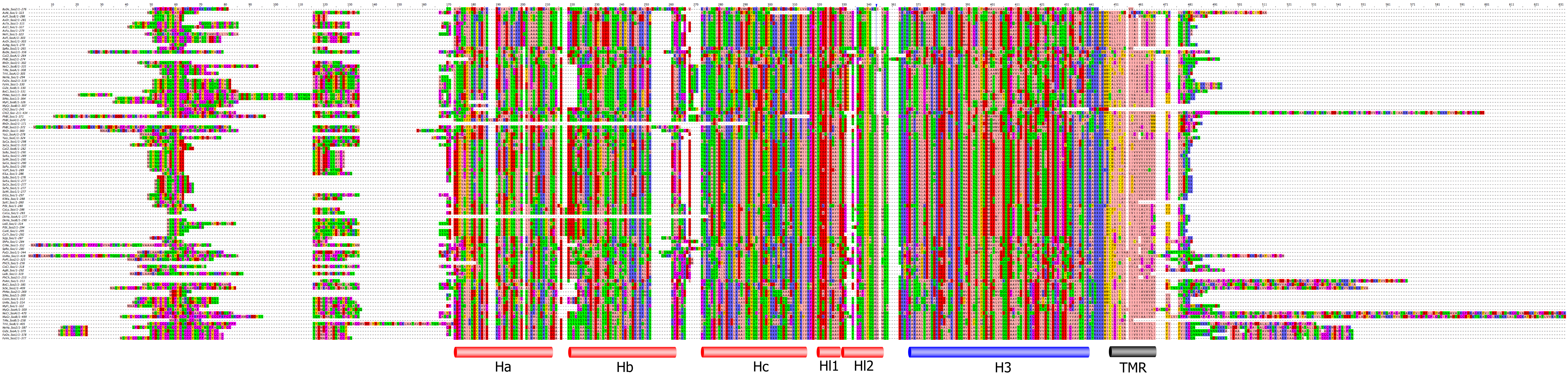
Ha
Hb
Hc
Hl1
Hl2
H3
TMR

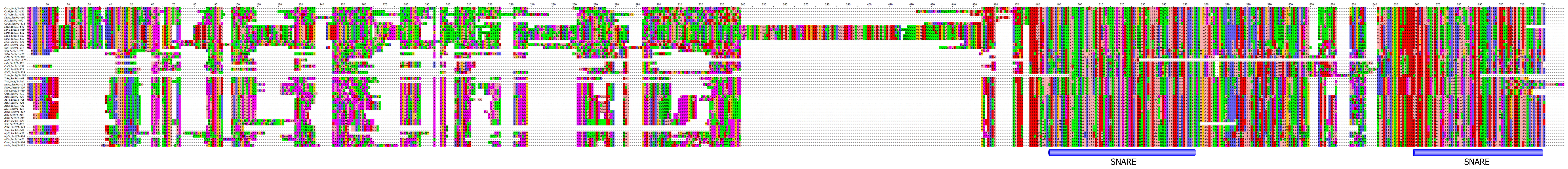
SNARE
SNARE

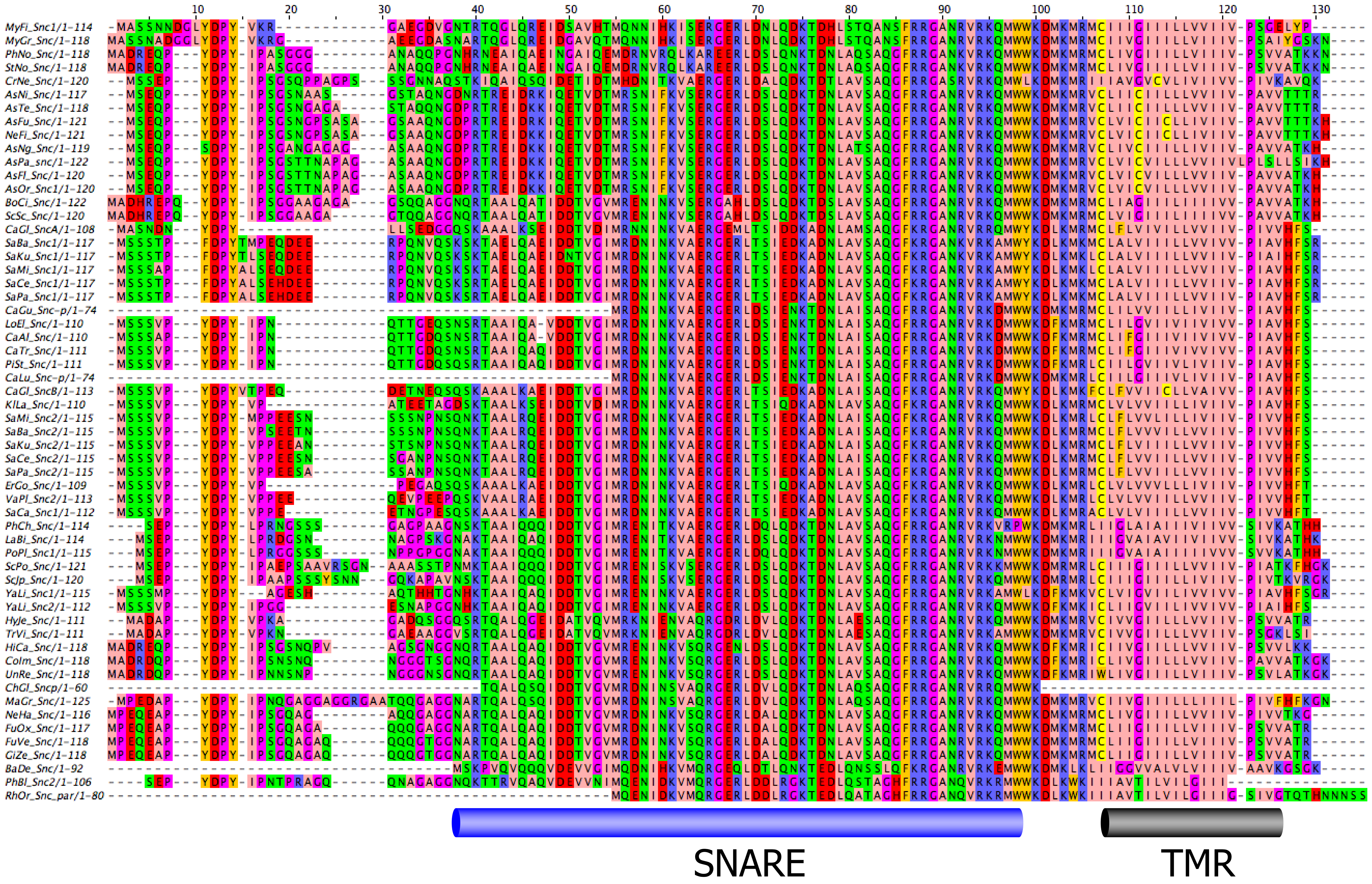

10 20 30 40 50 60 70 80 90 100 110 120 130
MyFi_Snc1/1-114
MyGr_Snc1/1-118
PhNo_Snc1/1-118
StNo_Snc1/1-118
CrNe_Snc1/1-120
AsNi_Snc1/1-117
AsTe_Snc1/1-118
AsFu_Snc1/1-121
NeFi_Snc1/1-121
AsNg_Snc1/1-119
AsPa_snc1/1-122
AsFl_Snc1/1-120
AsOr_Snc1/1-120
BoCi_Snc1/1-122
ScSc_Snc1/1-120
CaGl_SncA/1-108
SaBa_Snc1/1-117
SaKu_Snc1/1-117
SaMi_Snc1/1-117
SaCe_Snc1/1-117
SaPa_Snc1/1-117
CaGu_Snc-p/1-74
LoEl_Snc/1-110
CaAl_Snc/1-110
CaTr_Snc/1-111
PiSt_Snc/1-111
CaLu_Snc-p/1-74
CaGl_SncB/1-113
KlLa_Snc/1-110
SaMi_Snc2/1-115
SaBa_Snc2/1-115
SaKu_Snc2/1-115
SaCe_Snc2/1-115
SaPa_Snc2/1-115
ErGo_Snc/1-109
VaPl_Snc2/1-113
SaCa_Snc1/1-112
PhCh_Snc/1-114
LaBi_Snc/1-114
PoPl_Snc1/1-115
ScPo_Snc/1-121
ScJp_Snc/1-120
YaLi_Snc1/1-115
YaLi_Snc2/1-112
HyJe_Snc/1-111
TrVi_Snc/1-111
HiCa_Snc/1-118
CoIm_Snc/1-118
UnRe_Snc/1-118
ChGl_Sncp/1-60
MaGr_Snc/1-125
NeHa_Snc/1-116
FuOx_Snc/1-117
FuVe_Snc/1-118
GiZe_Snc/1-118
BaDe_Snc/1-92
PhBl_Snc2/1-106
RhOr_Snc_par/1-80
SNARE
TMR

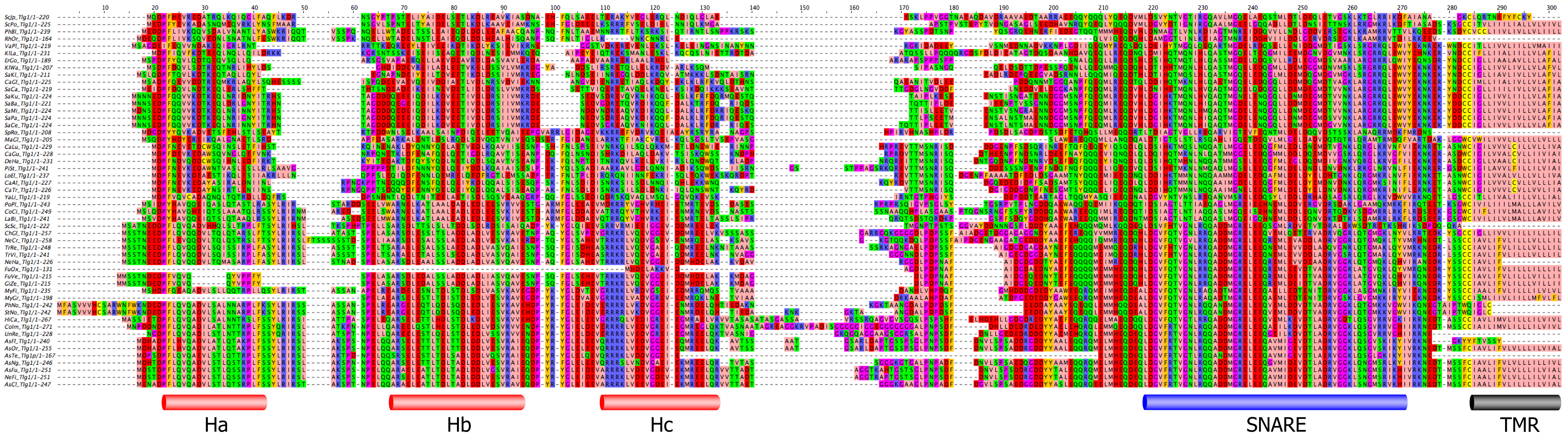
Ha
Hb
Hc
SNARE
TMR

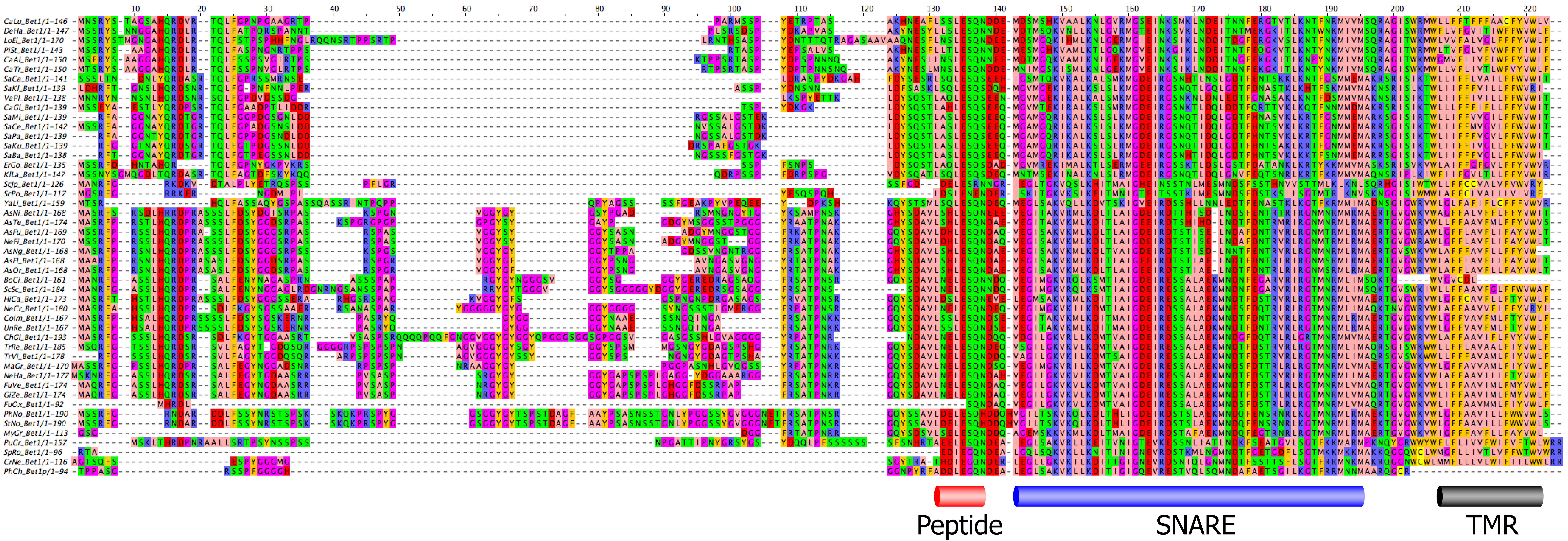
Peptide
SNARE
TMR

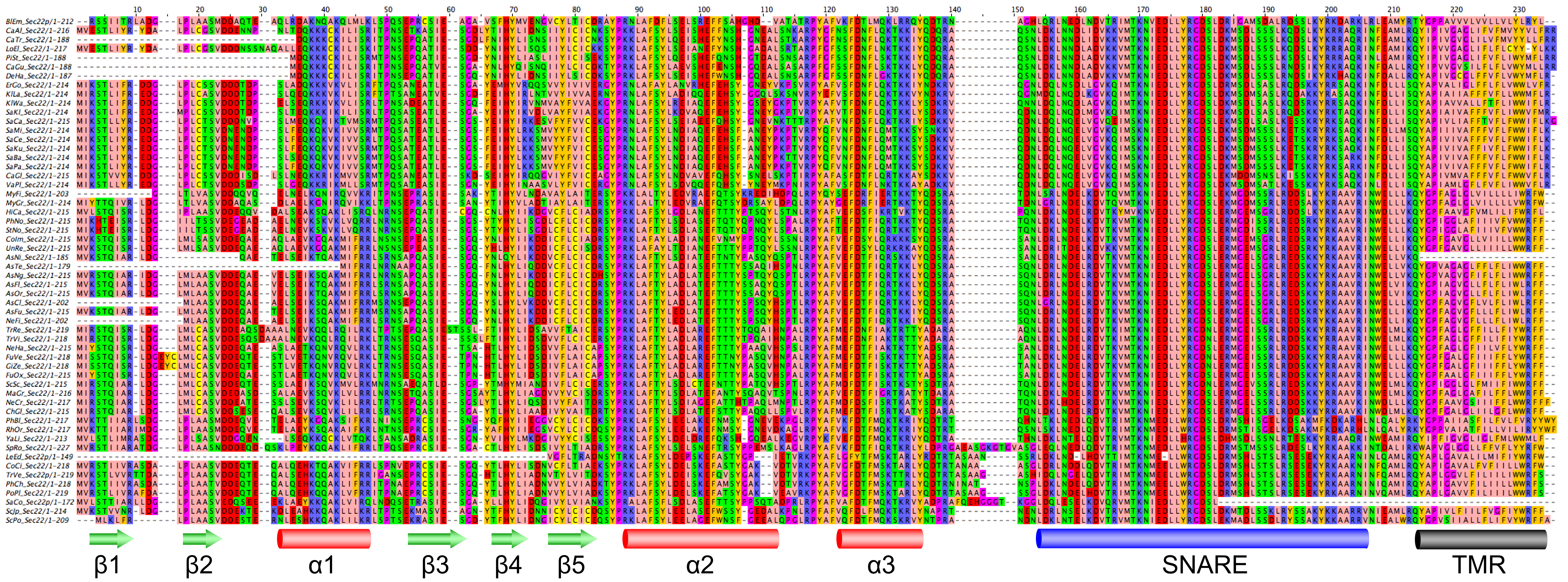
β1
β2
α1
β3
β4
β5
α2
α3
SNARE
TMR

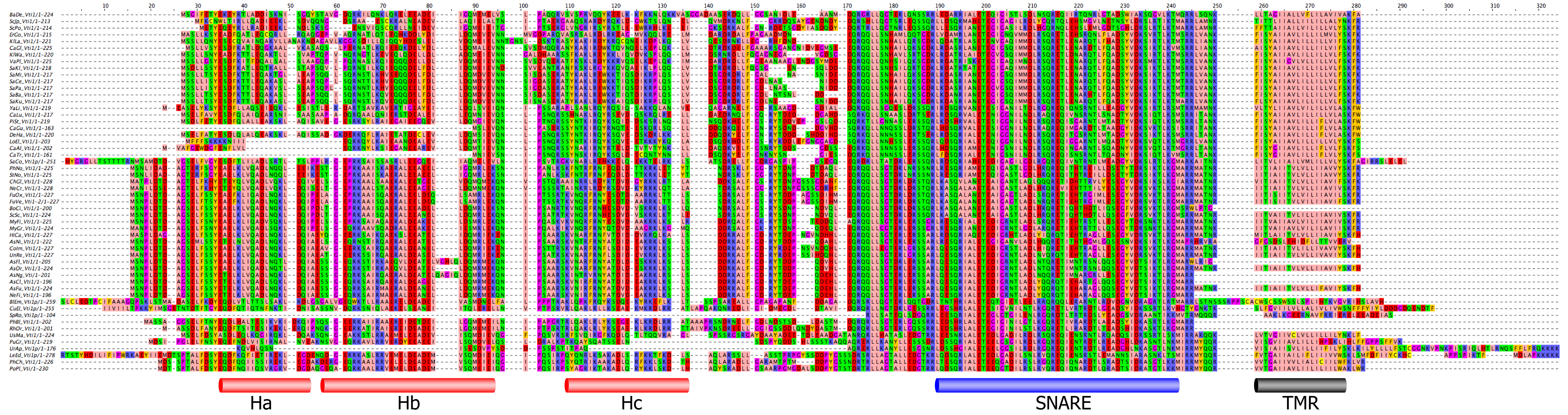
Ha
Hb
Hc
SNARE
TMR
